# Molecular phylogenetic, population genetic and demographic studies of *Nodularia douglasiae* and *Nodularia breviconcha* based on *CO1* and *16S rRNA*

**DOI:** 10.1038/s41598-020-72015-5

**Published:** 2020-10-06

**Authors:** Eun Hwa Choi, Gyeongmin Kim, Seung Hyun Cha, Jun-Sang Lee, Shi Hyun Ryu, Ho Young Suk, Young Sup Lee, Su Youn Baek, Ui Wook Hwang

**Affiliations:** 1 Department of Biology Education, Teachers College and Institute for Phylogenomics and Evolution, Kyungpook National University, Daegu, 41566 Republic of Korea; 2 Institute for Korean Herb-Bio Convergence Promotion, Kyungpook National University, Daegu, 41566 Republic of Korea; 3 School of Life Sciences, Graduate School, Kyungpook National University, Daegu, 41566 Republic of Korea; 4 Department of Life Sciences and Biotechnology, College of Natural Sciences, Soonchunhyang University, Asan, Chungcheongnam-do 31538 Republic of Korea; 5 Freshwater Biodiversity Research Division, Nakdonggang National Institute of Biological Resources, Sangju, Gyeongsangbuk-do 37242 Republic of Korea; 6 Department of Life Sciences, Yeungnam University, Gyeongsan, Gyeongsangbuk-do 38541 Republic of Korea

**Keywords:** Evolution, Genetics

## Abstract

Freshwater mussels belonging to the genus *Nodularia* (Family Unionidae) are known to be widely distributed in East Asia. Although phylogenetic and population genetic studies have been performed for these species, there still remain unresolved questions in their taxonomic status and biogeographic distribution pathways. Here, the nucleotide sequences of *CO1* and *16S rRNA* were newly determined from 86 N*. douglasiae* and 83 N*. breviconcha* individuals collected on the Korean Peninsula. Based on these data, we revealed the following results: (1) *N. douglasiae* can be divided into the three genetic clades of A (only found in Korean Peninsula), B (widely distributed in East Asia), and C (only found in the west of China and Russia), (2) the clade A is not an independent species but a concrete member of *N. douglasiae* given the lack of genetic differences between the clades A and B, and (3) *N. breviconcha* is not a subspecies of *N. douglasiae* but an independent species apart from *N. douglasiae*. In addition, we suggested the plausible scenarios of biogeographic distribution events and demographic history of *Nodularia* species.

## Introduction

Freshwater mussels belonging to the family Unionidae (Unionida, Bivalvia) consist of 753 species distributed over a wide area of the world encompassing Africa, Asia, Australia, Central and North America^1, 2^. The unionid mussels are known to play key roles in maintaining aquatic ecosystems such as nutrient circulation, water purification, and habitat supply^3^. Over the recent years, populations of unionid mussel species have rapidly been decreased due to global anthropogenic disturbances including desertification, water pollution, and river fragmentation^4, 5^. A total of 247 unionid species are now identified from the IUCN Red list of Threatened species^6, 7^. Interest in the taxonomic system and evolutionary relationships of unionids began with increasing reports about the fauna, ecological significance, and endangered status of freshwater mussels in North America and Europe since the late of twentieth century century^5, 8, 9^. In East Asia, ecological and evolutionary studies of unionids are still scarce though numerous species are currently at the blink of extinction in this taxon^10, 11^.

Unionid species survive parasitically on the scales, gills and fins of various fish species passing by in the first larval stage for several weeks or a month, fall off as juveniles, and grow burying themselves in the sediments or sticking to the crevice of rocks^12, 13^. Given the unique life history of the parasitic larval and adherent adult periods of this taxon, the biogeographic distribution patterns and genetic diversity of current species can reflect the history of past freshwater system formation and geological fluctuations^12, 13^. The Korean Peninsula is located at the end of East Asia. The seas between China and Japanese Archipelago were the lakes during the last glacial maximum which later served as the origin of the freshwater systems on the Korean Peninsula^14–16^. A tremendous endemism is observed on this peninsula, regardless of taxa, because many organisms that escaped glaciers could settle in countless refugia that had been formed due to complex terrain on this peninsula and have been speciated in the processes of isolation and adaptation^16–19^. Despite the biogeographical importance of the Korean Peninsula, the researches on the biogeographic origins and genetic diversity of unionids have been conducted insufficiently^5, 10^.

The *Nodularia* species are widely distributed throughout East Asia including the Korean Peninsula, China and Japanese Archipelago as well as Russia and Vietnam^9, 20^. *N. douglasiae* and *N. breviconcha* are only known species on the Korean Peninsula (Supplementary Fig. S1), and these two species were classified based on the morphological characteristics such as the size and inner epidermis color of the shell^21^. *N. douglasiae* has a bigger shell size than *N. breviconcha*. *N. douglasiae* is widely distributed throughout East Asia, while *N. breviconcha* is known to be a native species inhabiting only the Korean Peninsula^11, 22^. *N. breviconcha* had previously been treated as a subspecies of *N. douglasiae*^21, 23^, but it was recently revealed that these two species may be genetically and phylogenetically separated from each other^10, 12, 20, 24^. However, the studies conducted so far may not be enough to address the taxonomic status of *N. breviconcha* because they only used a small number of individuals from very limited sampling areas^10, 12, 20^. Further intensive studies are still required with more extensive sampling to clarify the taxonomic boundaries of this species.

Regarding the taxonomic status of *N. douglasiae*, Lopes-Lima et al*.*^10^ recently suggested that South Korean *N. douglasiae* should be divided into two species of *N. douglasiae* and *Nodularia* sp.1 based on *CO1* sequence difference. Although they attempted a sophisticated phylogeographic analysis on *Nodularia*, their study was not based on extensive sampling and population structure of *N. douglasiae* inhabiting the Korean Peninsula. The detailed population genetic structure of *N. douglasiae* in the Korean Peninsula should be uncovered with abundant sample collections, given that this peninsula would have been the most important intermediate link in shaping the contemporary distribution of this genus around the East Asia.

In this study, the phylogenetic relationships and population genetic structures of the East Asian *Nodularia* species were examined with mitochondrial *CO1* and *16S rRNA* data newly obtained from 86 N*. douglasiae* and 83 N*. breviconcha* individuals inhabiting the freshwater systems on the Korean Peninsula. The present analysis covers most of the areas inhabited by the two species on the Korean Peninsula and is based on extensive sampling to accurately determine the distribution of genetic variation, which was not expected from the previous studies^4, 10, 12, 20^. The obtained data here were applied to estimate and discuss the historical origin and distribution processes of *Nodularia* across East Asia by integrating with all previously known data. The present study can address the following four questions. First, are *N. douglasiae* and *N. breviconcha* distinctly separated to the extent of species level? Second, in *N. douglasiae* inhabiting the Korean Peninsula, is there a genetic lineage that is distinct enough to be considered a new *Nodularia* species? Third, what biogeographic pathways did *Nodularia* species inhabiting the Korean Peninsula come through? Finally, is there any pattern of genetic differentiation among the populations of *N. douglasiae* and *N. breviconcha* reflecting the known historical events of freshwater system formation and fluctuations?

## Results

### Sequence analysis of CO1 and 16S rRNA

**Figure 1:**
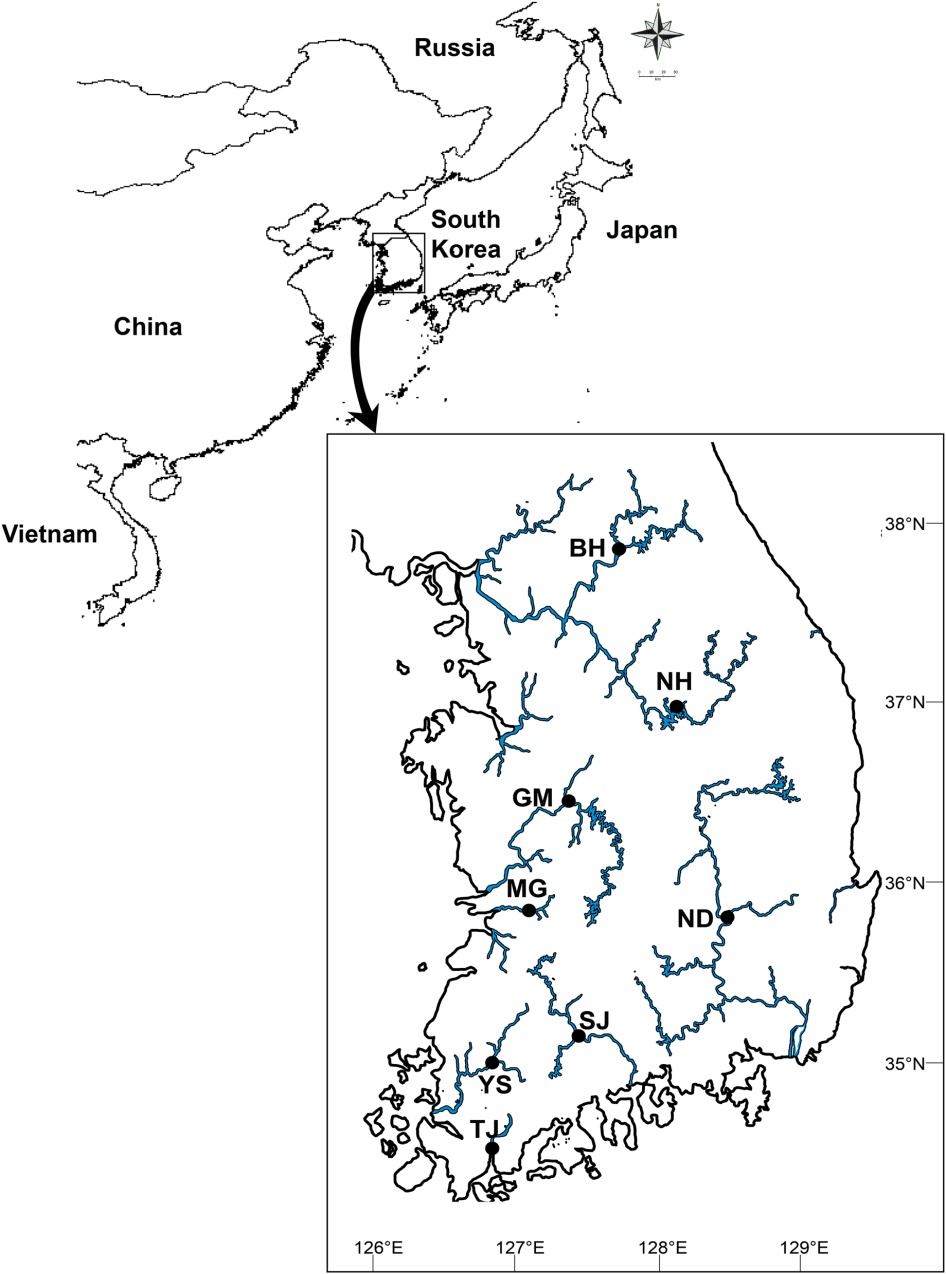
Sampling sites for 86 *Nodularia douglaisae* and 83 *Nodularia breviconcha* in the Korean Peninsula. The sampling locations of the seven *N. douglasiae* populations (BH, GM, MG, ND, SJ, YS, and TJ) and the six *N. breviconcha* populations (BH, NH, GM, SJ, YS, and TJ) were depicted on the map. For the comparison *Nodularia* species inhabiting in East Asian countries, we used the data from Japan, China, Russia, and Vietnam as well as the Korean Peninsula. The maps were generated by Adobe Illustrator CC 2020 using a GIS shape file retrived from administrative areas database in DIVA GIS (https://www.diva-gis.org/).

The mitochondrial *CO1*, which is 524 bp in length, was amplified and sequenced from 86 individuals of *N. douglasiae* and 83 of *N. breviconcha* collected from seven and six rivers, respectively, on the Korean Peninsula (Table 1, Fig. 1). Extensive sampling was performed in populations BH, GM, and ND of *N. douglasiae*, and in populations BH and NH of *N. breviconcha.* We had trouble collecting individuals from the other populations, due to the scarcity, and ended up sampling only one to five individuals per population. In our analysis, 25 and 16 *CO1* haplotypes were detected from *N. douglasiae* and *N. breviconcha*, respectively (Supplementary Tables S1 and S2). No haplotype was interspecifically shared. A total of 30 and 21 polymorphic sites of *N. douglasiae* and *N. breviconcha* were identified, respectively, of which eight and nine were singleton variable sites, and 22 and 12 were parsimoniously informative sites. As shown in Table 1, in *N. douglasiae*, the number of *CO1* haplotypes per population varied from one (SJ, TJ, and YS) to 13 (ND). Haplotype diversity was highest in the population ND (*h* = 0.878). The population ND only showed the statistically significant negative Fu's *F*s value, which could be a signature of a sudden historical population expansion. In *N. breviconcha*, the number of *CO1* haplotypes per population was higher in BH (6) and NH (7) than the others. Haplotype diversity was relatively high in BH (*h* = 0.695) and SJ (*h* = 1; but estimated from only two individuals). *N. breviconcha* showed statistically significant negative Fu's *F*s value (− 5.203) when considering all observed haplotypes.

**Table 1:** Genetic diversity indices of the *CO1* and *16S rRNA* haplotypes obtained from 86 individuals of *Nodularia douglasiae* and 83 of *Nodularia breviconcha* on the Korean Peninsula.

Species	Tributary	Pop	*CO1*									*16S rRNA*								
			*N*	*N*_1_	*N*_H_	*N*_P_	*h*	*S*	*π*	Tajima's *D*	Fu's *F*s	*N*	*N*_1_	*N*_H_	*N*_P_	*h*	*S*	*π*	Tajima's *D*	Fu's *F*s
*N. douglasiae*	Bukhan	BH	16	16	3	2	0.508	5	0.003	0.058	2.039	16	16	3	1	0.567	2	0.002	0.555	0.348
	Geum	GM	26	26	7	5	0.708	20	0.015	1.610	4.916	26	26	9	5	0.794	11	0.007	− 0.415	− 1.642
	Mangyeong	MG	5	5	3	2	0.800	5	0.005	1.124	1.220	5	*–*	*–*	*–*	*–*	*–*	*–*	*–*	*–*
	Nackdong	ND	30	30	13	11	0.878	17	0.006	− 0.874	− 3.887*	30	30	8	6	0.593	10	0.003	− 1.653*	− 3.171*
	Seomjin	SJ	5	5	1	0	0.000	0	0.000	nd	nd	5	3	1	0	0.000	0	0.000	nd	nd
	Tamjin	TJ	3	3	1	1	0.000	0	0.000	nd	nd	3	3	1	0	na	na	na	na	na
	Yeongsan	YS	1	1	1	1	na	na	na	na	na	1	1	1	0	0.000	0	0.000	nd	nd
	**Total**		86	86	25	22	0.918	30	0.013	0.492	− 3.159	86	79	16	12	0.834	18	0.00656	− 1.055	− 5.164*
*N. breviconcha*	Bukhan	BH	29	29	6	3	0.695	6	0.002	− *1.050*	− *1.785*	29	29	4	3	0.197	2	0.000	− 1.249	− 1.628
	Geum	GM	1	1	1	1	na	na	na	na	na	1	*–*	*–*	*–*	*–*	*–*	*–*	*–*	*–*
	Namhan	NH	43	43	7	4	0.563	9	0.002	− *1.415*	− *1.985*	43	43	3	2	0.092	4	0.001	− 1.873*	− 3.324**
	Seomjin	SJ	2	2	2	1	1.000	2	0.004	nd	*0.693*	2	1	1	1	na	na	na	na	na
	Tamjin	TJ	5	5	2	2	0.400	1	0.001	− *0.817*	*0.090*	5	4	1	0	0.000	0	0.000	nd	nd
	Yeongsan	YS	3	3	2	1	0.667	1	0.001	nd	*0.201*	3	2	1	0	0.000	0	0.000	nd	nd
	**Total**		83	83	20	12	0.741	21	0.001	− 1.372	− 5.203*	86	79	5	6	0.146	4	0.000	− 1.684**	− 5.345***

This table includes population codes (Pop), the number of samples (*N*), the number of samples from which PCR fragments were obtained (*N*_1_) the number of haplotypes (*N*_H_), the number of private haplotypes (*N*_P_), haplotype diversity (*h*), the number of segregating sites (*S*), and nucleotide diversity (*π*). Significant value: Bold (*: *p* < 0.05, **: *p* < 0.01), not significant value: Italic, *na* not available, *nd* not determined.

The mitochondrial *16S rRNA* (357 bp long) was successfully amplified and sequenced from 79 out of 86 individuals of *N. douglasiae* and 79 out of 83 individuals of *N. breviconcha* (Fig. 1, Table 1), which yielded 16 haplotypes for *N. douglasiae* and five for *N. breviconcha* (Supplementary Tables S3 and S4). No haplotype was interspecifically shared in *16S rRNA*. A total of 18 and five polymorphic sites from *N. douglasiae* and *N. breviconcha* were identified, respectively, of which ten and two were singleton variable sites, and 8 and 3 parsimoniously informative sites. In *N. douglasiae*, relatively high haplotype diversities were found in populations BH, GM, and ND because of limited sampling in the other populations (Table 1). The highest haplotype diversity (*h* = 0.794) and nucleotide diversity were observed in population GM. Only population ND showed significant negative Tajima's *D* and Fu's *Fs*, indicating a drastic population expansion. In *N. breviconcha*, only population NH showed significant negative Tajima’s *D* and Fu’s *F*s, which is also the signature of demographic expansion. Populations BH (though statistically insignificant) and NH of *N. breviconcha* consistently showed negative Tajima’s *D* and Fu’s *F*s like in *CO1*, strongly implying historical expansions of these populations.

### Phylogenetic and population analyses

**Figure 2:**
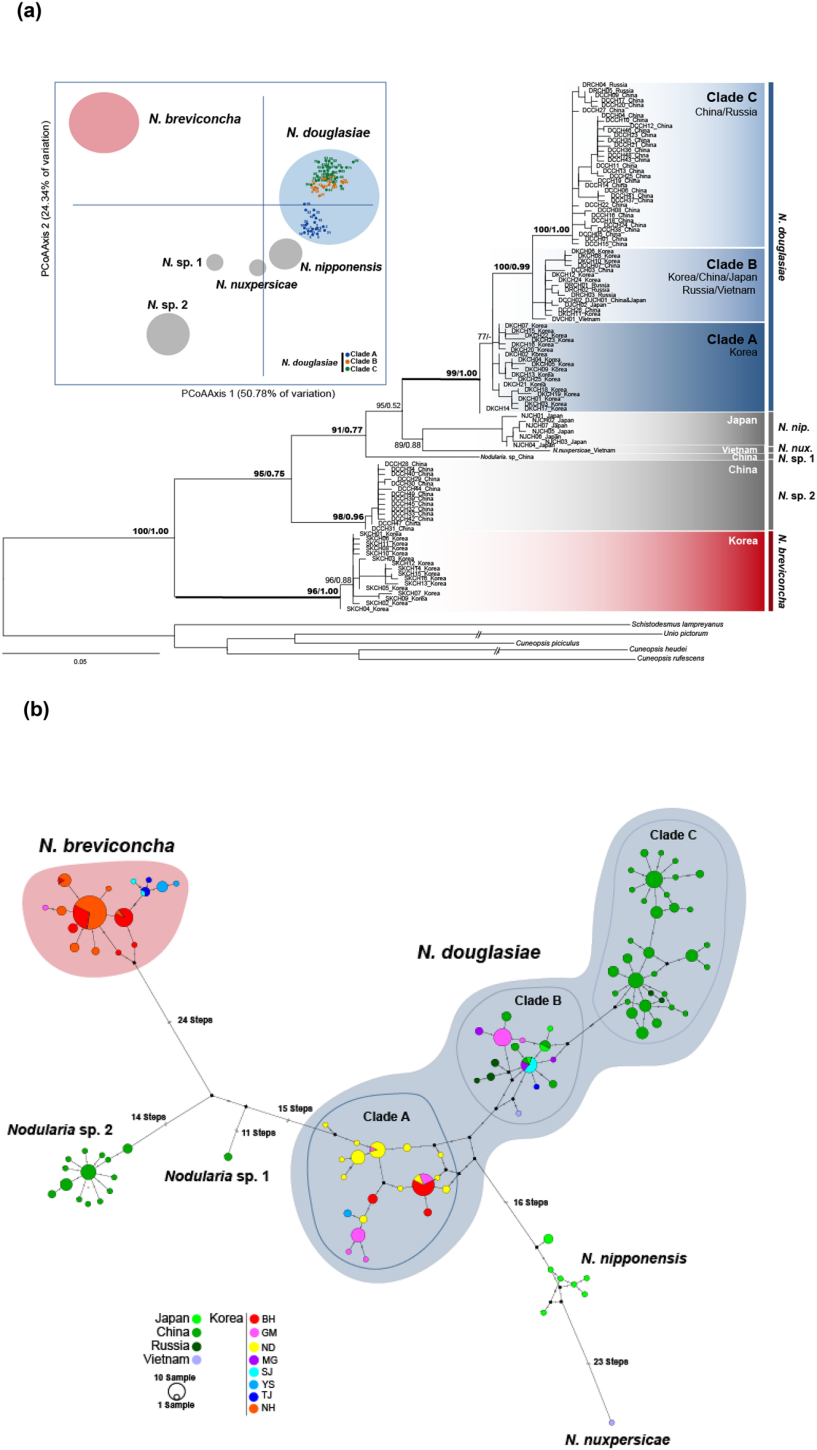
(**a**) Phylogenetic tree reconstructed using 106 *CO1* haplotypes of six *Nodularia* species based on Bayesian inference (BI) and maximum likelihood (ML) methods*.* The tree was reconstructed using 67 *CO1* haplotypes of *N. douglasiae*, 16 of *N. breviconcha*, one of *N.* sp.1, 14 of *N.* sp.2, seven of *N. nipponensis*, and one of *N. nuxpersicae.* Five species were used as outgroups. The species used in this phylogenetic analyses were listed in Supplementary Tables S5 and S7. The numbers on branches indicate bootstrap values (ML) and posterior probabilities (BI) being separated by slash. The PCoA graph was generated based on the *CO1* haplotypes from six *Nodularia* species. (**b**) The unrooted TCS haplotype network was constructed based on the *CO1* haplotypes from the six *Nodularia* species. Haplotype frequency is related to the size of the circle. Different colors within the nodes refer to different sampling sites shown in Fig. 1. TCS network was generated using PopART (https://popart.otago.ac.nz/index.shtml) and then modified by Adobe Illustrator CC 2020.

For the present phylogenetic and population analyses based on *CO1* haplotypes, we retrieved and aligned additional genetic information from NCBI GenBank database including 42 *CO1* haplotypes from 108 sequences for *N. douglasiae*, one haplotype from 2 sequences for *Nodularia* sp.1, 14 haplotypes from 26 sequences for *Nodularia* sp.2, 7 haplotypes from 9 sequences for *N. nipponensis*, and one haplotype from one sequence of *N. nuxpersicae* (Supplementary Table S5). Five haplotypes for five outgroup species were also retrieved. With 25 haplotypes for *N. douglasiae* and 16 haplotypes for *N. breviconcha* newly obtained in this study, a total of 106 *Nodularia CO1* haplotypes were used for the phylogenetic and population genetic analyses. According to the ML and BI phylogenetic trees reconstructed with 106 *Nodularia CO1* haplotypes (Fig. 2a), *N. douglasiae* (BP 99% and BPP 1.00) and *N. breviconcha* (BP 96% and BPP 1.00) formed independent monophyletic groups within the clade of the genus *Nodularia* with high node confidence values, respectively. These two species were clearly separated by other intrageneric species, *N. nipponensis*, *N. nuxpersicae*, *N.* sp.1, and *N.* sp.2. *N. douglasiae* was directly clustered with *N. nipponensis* and *N. nuxpersicae*, with *N. breviconcha* being placed at the basal node. Within the group of *N. douglasiae*, there were three genetic lineages of the clades A, B, and C, though the clade A was paraphyletic to the clade B. In the results of the PCoA and TCS network analyses (Fig. 2; Supplementary Fig. S2a), *N. douglasiae* was evidently separated from *N. breviconcha* as shown in the phylogenetic analyses (Fig. 2a). In the PCoA (Fig. 2a), clades B and C of *N. douglasiae* were almost overlapped with each other, though clade A was slightly distant from clades B and C. In the TCS network (Fig. 2b), the three clades were also closely related with small number of mutation steps.

As shown in Fig. 2, the clade A consisted of the haplotypes of *N. douglasiae* only from BH, ND, YS, and GM populations in the Korean Peninsula, the clade B included the haplotypes from relatively wide distribution range including northeast China, west Japan, Russia, Vietnam, as well as South Korea (GM, MG, SJ and TJ). On the other hand, the haplotypes of the clade C were only from west China and Russia. Thus, *CO1* haplotypes of *N. douglasiae* collected from the Korean Peninsula could be allocated into clades A and B; no clade C was not found in this peninsula. Also, in population GM, haplotypes from the clades A and B coexisted.

**Figure 3:**
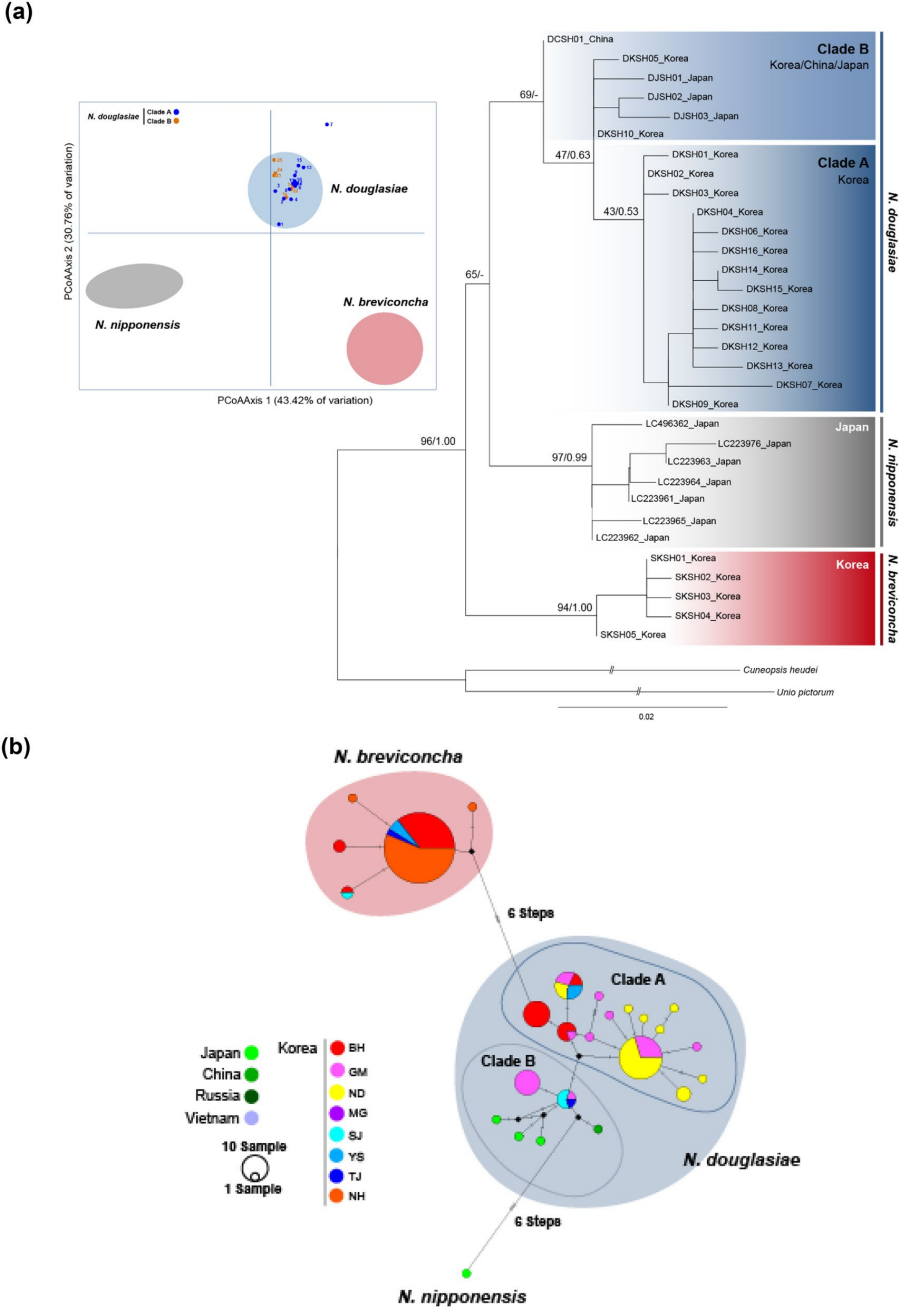
(**a)** Phylogenetic tree reconstructed based on the maximum likelihood (ML) and Bayesian inference (BI) methods using *16S rRNA* haplotypes of the three *Nodularia* species. The tree is constructed using *16S rRNA* haplotypes of 20 N*. douglasiae* and five of *N. breviconcha* and seven of *N. nipponensis*. Two species were used as outgroups. The species used in this phylogenetic analysis were listed in Supplementary Tables S6 and S8. The numbers on branches indicate the bootstrap values (ML) and posterior probabilities (BI) being separated by slash. The PCoA graph was generated based on the *16S rRNA* haplotypes from the three *Nodularia* species showed on phylogenetic tree. (**b**) The unrooted TCS haplotype networks was constructed based on the haplotypes of *16S rRNA* haplotypes from the three *Nodularia* species. Haplotype frequency is related to the size of the circle. Different colors within the nodes refer to different sampling sites shown in Fig. 1. TCS network was generated using PopART (https://popart.otago.ac.nz/index.shtml) and then modified by Adobe Illustrator CC 2020.

Based on the haplotypes of *16S rRNA*, the ML and BI trees (Fig. 3a) showed that *N. breviconcha* (BP 94% and BPP 1.00) and *N. nipponensis* (BP 97% and BPP 0.99) formed strong monophyletic groups, though the monophyly of *N. douglasiae* (BP 69% in ML) was supported with relatively lower node confidence value. *N. nipponensis* appeared to be a sister species to *N. douglasiae*. Overall, tree topology inferred from *16S rRNA* was consistent with that reconstructed based on *CO1* data (Fig. 2a). *N. douglasiae* and *N. breviconcha* formed independent monophyletic groups. Due to the missing of Russian samples and the limited number of Chinese haplotypes, the clade C shown in *CO1* data was not observed in *N. douglasiae*. The monophyly of clade A was weakly supported, and the clade B was paraphyletic to the clade A. According to the results of the PCoA and TCS network analyses based on *16S rRNA* (Fig. 3; Supplementary Fig. S2b), *N. douglasiae* was evidently separated from *N. breviconcha*, just as observed in the *CO1* results (Fig. 2; Supplementary Fig. S2a). The clades A and B of *N. breviconcha* appeared to be almost completely overlapped with each other in the PCoA (Fig. 3a; Supplementary Fig. S2b), with these two being separated by only a single mutation step in TCS network (Fig. 3b).

### Demographic analysis

**Figure 4:**
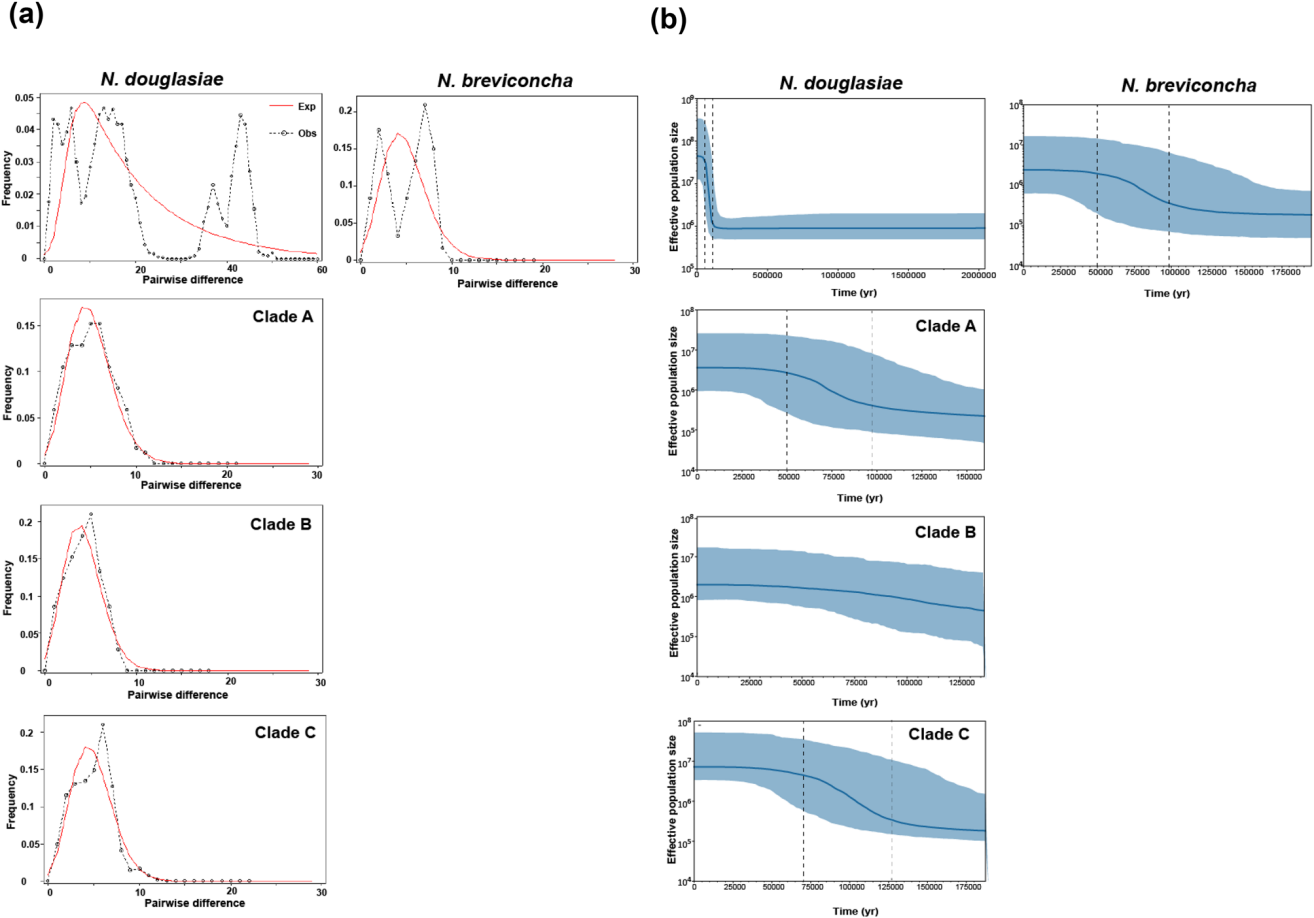
(**a**) The mismatch distribution analysis (MDA) and (**b**) Bayesian skyline plot (BSP) analysis estimated based on the *CO1* haplotypes of *Nodularia douglasiae,* the three *N. douglasiae* clades of A, B, and C*,* and *Nodularia breviconcha*. The 67 *CO1* haplotypes for *N. douglasiae* (Supplementary Table S7), and 16 haplotypes for *N. breviconcha* (Supplementary Table S2) were employed. The number of haplotypes for the clades of *N. douglasiae* refers to Table 2.

The neutrality tests were performed with *CO1* and *16S rRNA* haplotypes of *N. douglasiae* and *N. breviconcha* (Table 2). All the three clades of *N. douglasiae* in the *CO1* data showed significant negative values in Fu’s *F*s, respectively, but not in Tajima’s *D*. When measured on the species itself, the same pattern was appeared. In *N. breviconcha*, both Tajima’s *D* and Fu’s *F*s values were significantly negative. On the other hand, based on the 16*S rRNA* data, both *N. douglasiae* and *N. breviconcha* showed significant negative values only in Fu’s *F*s, respectively, but not in Tajima’s *D*s. When a mismatch distribution analyses (MDA) based on only *CO1* were performed for each species, both *N. douglasiae* and *N. breviconcha* (Fig. 4a) showed multimodal curves. When analyzed for each clade of *N. douglasiae* (Fig. 4a) a unimodal curve was observed.

**Table 2:** The results of the neutrality tests with *CO1* and *16S rRNA* for *Nodularia douglasiae* and *Nodularia breviconcha* on the Korean Peninsula.

Gene	Species	Haplotype no.^a^	Detailed clade	Tajima’s *D*	Fu’s *F*s
*CO1*	*N. douglasiae*	19	Clade A	− 0.897	− 17.712***
		15	Clade B	− 1.444	− 13.424***
		33	Clade C	− 1.464	− 25.559***
		67	Clade A + Clade B + Clade C	− 1.120	− 24.401***
	*N. breviconcha*	16	*–*	− 2.187**	− 13.506***
*16S rRNA*	*N. douglasiae*	20	*–*	− 1.539	− 22.986***
	*N. breviconcha*	5	*–*	− 1.124	− 3.068**

^a^The detail information of the numbers of haplotypes and employed individuals refer to Supplementary Tables S5–S8.

Bayesian skyline plot (BSP) analyses based on the *CO1* data were performed to examine the pattern of fluctuation in effective population size for *N. douglasiae*, and *N. breviconcha* (Fig. 4b), and each of the three clades of *N. douglasiae* (Fig. 4b). The effective population size of *N. douglasiae* was dramatically increased from ca. 100 kya but was ceased at around ca. 50 kya. *N. breviconcha* began to gradually grow from ca. 100 kya, which was ceased at round ca. 50 kya. Among the three clades within *N. douglasiae*, a noticeable growth event was observed only in A and C at a certain period. Demographic expansion occurred slightly earlier in the clade C (ca. 100 – 50 kya) than A (ca. 130 –70 kya).

### Divergence time estimation

**Figure 5:**
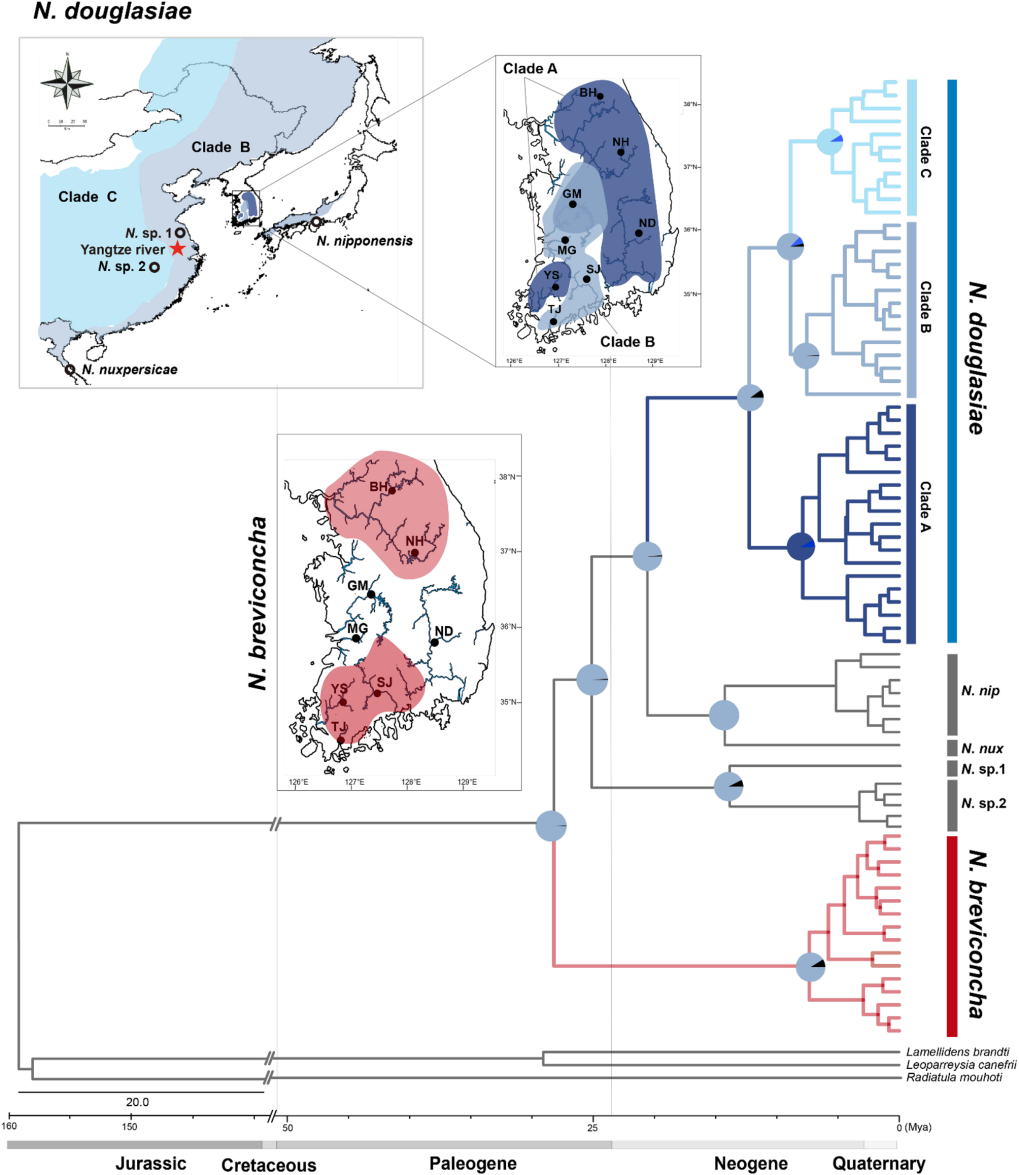
Time-calibrated Bayesian tree reconstructed with 106 CO1 haplotypes of six *Nodularia* species using the BEAST 2.6.0. program with the inference of ancestral areas under Bayesian binary MCMC (BBM) model implemented in the RASP 3.2 program. Ancestral areas were hypothesized based on the distribution ranges of each *Nodulari*a species identified in the phylogenetic tree, which were shown as the format of pie chart on the nodes. See Fig. 1 for the details about how to generate the map.

**Figure 6:**
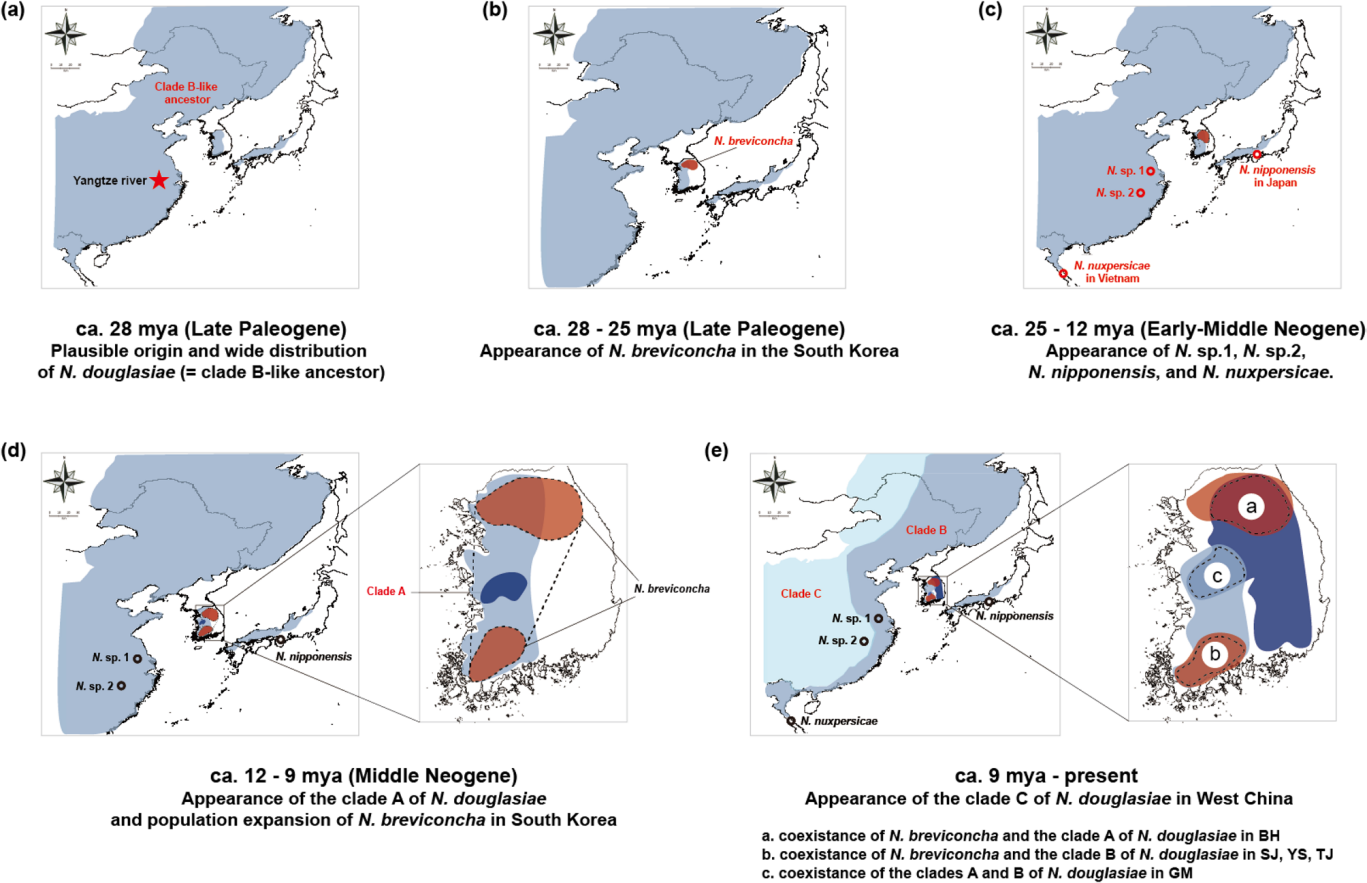
The plausible scenario of changes of biogeographical distribution of the six *Nodularia* species in East Asia, focusing on *Nodularia douglasiae* and *Nodularia breviconcha* in the Korean Peninsula. See Fig. 1 for the details about how to generate the map.

According to the molecular clock analysis by the BEAST program (Supplementary Fig. S3), it is estimated that *N. breviconcha* and the other examined *Nodularia* species shared a common ancestor at about 28.21 mya. The divergence time of *N. douglasiae* and *N. nipponensis* was estimated to be around 20.57 mya. Within *N. douglasiae*, clade A was first diverged off about 12.26 mya, and then the clades B and C were diverged from each other at around 8.88 mya. The S-DIVA analysis under a Bayesian Binary Markov Chain Monte-Carlo (BBM) model (Fig. 5) indicated that a hypothetical common ancestor of the six examined *Nodularia* species originated from around Chinese Yangtze River where the clade B haplotypes are dominantly observed. This result may also indicate that the region may be regarded as a plausible origin of all examined *Nodularia* species in East Asia, with raising the possibility of the clade B as ancestral haplotypes (Fig. 6; Supplementary Fig. S4).

## Discussion

This study was designed to provide a taxonomic and phylogenetic revision of the species in the genus *Nodularia* distributed throughout northeastern Asia (Beringia, Amur-Korea, Japan-Skalin, and Eastern China) with a focus on *N. douglasiae* and *N. breviconcha*. Although *N. breviconcha* is now known as a native species endemic to South Korea, it has often been treated as a subspecies of *N. douglasiae*, even after the start of extensive taxonomic study for *Nodularia* species^20, 21^. It has been consistently pointed out that these two species have distinct morphological differences^21^. Recent studies based on *CO1* sequences^10, 12, 20^ have insisted that *N. breviconcha* should be considered as an independent species apart from *N. douglasiae* on the basis of the remotely related phylogenetic relationships between the two species within the genus *Nodularia*. Lopes-Lima et al*.*^10^ gave a new scientific name to this species as *N. breviconcha*. However, recent taxonomic and phylogenetic studies on the genus *Nodularia* have an obvious drawback, limited sampling for *N. breviconcha*. For examples, Klishko et al*.*^20^ used only a single individual, and Lopes-Lima et al.^10^ considered 6 haplotypes for the analysis (individual information not shown). Therefore, more extensive sampling was needed to address the distribution of its intraspecific variation of *N. breviconcha*. Thereby, a total of 83 N*. breviconcha* and 86 N*. douglasiae* individuals were collected from the six and seven different drainages in South Korea, respectively, in the present study. With the collected samples, the nucleotide sequences of *CO1* and *16S rRNA* were determined, which were subjected to the subsequent population genetic and phylogenetic analyses.

Summarizing all the present results including the phylogenetic trees, PCoA, and TCS networks, it was rather ironic that there have been debates over the taxonomic status of *N. douglasiae* and *N. breviconcha*. Their genetic differentiation was highly evident. Thus, we here confirmed *N. breviconcha* to be an independent species. In the phylogenetic trees reconstructed using *CO1* and *16S rRNA*, *N. breviconcha* occupied a basal position in the clade of the genus *Nodularia* without exception. In addition, *N. nipponensis*, *N. nuxpersicae*, and *Nodularia* sp.1 and sp.2 were even more closely related to *N. douglasiae* than *N. breviconcha*. According to the BEAST analysis, *N. breviconcha* was first branched out and colonized in the freshwater systems of the Korean Peninsula during the late Paleogene (ca. 28 mya). The appearance of this new endemic species may be due to the adaptation to new habitats of the Korean Peninsula, markedly different from other parts of the East Asia. Given a much smaller shell size than *N. douglasiae*^21^, *N. breviconcha* may have occupied different types of niche while avoiding competition with *N. douglasiae*. No ecological studies have yet been conducted on the adaptation of these two species to their microhabitats on the Korean Peninsula so far.

In *N. breviconcha*, a star-like pattern appeared in the haplotype network without any strong genetic structure among populations, indicating that there was little spatial isolation process after geographical dispersion. However, the genetic structure was slightly formed in the *CO1* haplotype network. Moreover, the mismatch analyses (MDA) with *CO1* showed a multi-modal curve, suggesting the probability that two slightly different genetic groups existed within *N. breviconcha*. The results of examining both the Tajima’s *D* and Fu’s *F*s using *CO1* and *16S rRNA* at the species level clearly support the sudden demographic expansion of this species following colonization on the Korean Peninsula.

In the phylogenetic trees, PCoA, and TCS networks obtained from the *CO1* sequences, *N. douglasiae* haplotypes were assigned to the three clades of A, B, and C. With *16S rRNA* sequences, we could also reconfirm the structure of the clades A and B, though the existence of clade C was not confirmed as NCBI GenBank lacked *16S rRNA* information from Chinese and Russian specimens. In *CO1* sequences, clades B and C showed a star-like haplotype network pattern, unlike clade A, showing the signature of population expansion during the formation of frequent confluences in the geological period. The individuals belonging to the clade A are dominantly distributed in the Nakdong River (ND) and the upper reaches of the Han River (BH). Probably, clade A may not have the opportunity of demographic expansion in the process of dispersing along the limited habitat areas on the narrow peninsula. The MDA analysis based on *CO1* showed a more complicated multi-modal curve than that of *N. breviconcha*, but each of *N. douglasiae* clades showed a unimodal curve. Such pattern suggests the probability that no genetic structure existed within each clade, despite a definite genetic structure within the whole *N. douglasiae* clades. However, we could not find a possible signature of population expansion within whole *N. douglasiae* population, considering that statistically significant values were not observed in the Tajima’s *D* tests for *CO1* and *16S rRNA* at the species level and the clade level, though the Fu’s *F*s values were significant in both loci.

Lopes-Lima et al*.*^10^ recently suggested that the clade A inhabiting the Korean Peninsula should be assigned as *Nodularia* sp.1 with the possibility of being an independent species apart from *N. douglasiae*. However, this suggestion was made based on three different haplotypes. Our robust phylogenetic results reconstructed based on *CO1* and *16S rRNA* do not support their view of considering *N. douglasiae* as a species complex with multiple species candidates including the clade A. The clade A was not monophyletic but paraphyletic to the monoclade of B and C in our phylogenetic results. In addition, the degree of genetic differences between clades A and B was not enough to consider the level of interspecific difference in our haplotype network analyses. Clade A was overlapped with clade B in PCoA results obtained based on both *CO1* and *16S rRNA*.

According to the results of the molecular clock analysis using the BEAST program, clade A was first branched off from the ancestral *N. douglasiae* and colonized the Korean Peninsula around mid-Neogene (ca. 12 mya). Afterwards, it seems that clades B and C seem to be born around late Neogene (ca. 9 mya). The clade B of *N. douglasiae* consisted of the haplotypes from east China, west Japan, east Russia and Vietnam, as well as South Korea, whereas the clade C haplotypes were obtained from the samples of central China and Russian Far East. Through the S-DIVA analysis under BBM model, geographic locations of the most common ancestors were examined at each node of speciation of *Nodularia* and clade formation within *N. douglasiae*. The clade B haplotypes (= a plausible common ancestor of *Nodularia*) were dominantly located at the Yangtze River in China, and the clade A was diverged on the way of the eastward inflow into the Korean Peninsula. In addition, it is thought that the Korean Peninsula probably served as a bridgehead for the dispersal of clade B to west Japan.

As summarized in Fig. 6, we attempted to reconstruct the historical pathway of the two South Korean *Nodularia* species, *N. douglasiae* and *N. breviconcha*, to colonize the Korean Peninsula. Clade B haplotypes can be regarded as the potential origin of *N. douglasiae*, as shown in the S-DIVA results (B-like ancestor; Fig. 6a). The distribution of the clade B is concentrated in the eastern part of China and the western part of the Korean Peninsula, centered on the Yellow Sea^10, 12^. In particular, the clade B populations are abundantly found mainly in Hongze Lake, Taihu Lake and the surrounding freshwater systems^10^ that are thought to have been frequently connected to the Yangtze River, a long river system running through south China, along geological times^10^. The Yangtze River in China is the hotspot with the largest number of endemic freshwater mussel species in East Asia and is believed to have been the origin of the contemporary species diversity^11^. Taken together, it is likely that the present data supported the demographical history of *Nodularia* species that they were originated from the Yangtze River in China and spread to the East Asian freshwater systems including the Korean Peninsula and Japan (around 28 mya during the late Paleogene). After wide distribution of the clade B of *N. douglasiae*, it is likely that *N. breviconcha* endemic to South Korea was first raised possibly at the Han River including BH and NH during the late Paleogene (around 28–25 mya; Fig. 6b). The speciation events of *N.* sp.1 (east China)*, N.* sp.2 (east China), *N. nipponensis* (west Japan), and *N. nuxpersicae* (north Vietnam) might occur from the late Paleogene to the middle Neogene (around 25–12 mya; Fig. 6c). In the middle Neogene around 12–9 mya (Fig. 6d), it is likely that the clade A of *N. douglasiae* might have colonized the middle area (i.e. GM) of South Korea, while *N. breviconcha* seemed to expand its habitats into the southward of South Korea. From late Neogene (ca. 9 mya), it is thought that the clade C of *N. douglasiae* was raised in the west China or Russia and the clade A of *N. douglasiae* expanded to the eastward of South Korea (Fig. 6e). As shown in Fig. 6e, it is interesting that the distribution of *N. breviconcha* were overlapped with clade A (ⓐ; BH) and clade B (ⓑ; SJ, YS, and TJ). The distribution of clades A and B were also overlapped with each other (ⓒ; GM).

According to the present results, there still remain two big questions as follows. First, why is the clade A of *N. douglasiae* relatively rare in the west coastal rivers but overwhelming in the east areas of the Korean Peninsula, mainly in the Nakdong River and the upper reaches of Han River? Although the Han River including BH has likely been colonized through the paleo-Yellow River confluence, it is difficult to infer the biogeographic pathways that formed the Nakdong River population. Since continental shelf is not well-developed around the mouth of the Nakdong River, estuary coalescence with adjacent rivers would not have been possible^25–28^. If so, it is somewhat surprising that the genetic diversity of the Nakdong River population was relatively and significantly higher than the others in the present results, considering that this population would have flourished via the small-scale migration likely with watershed capture^26^. Given the high genetic diversity, there is no other way than to assume that there has been a persistent gene flow elsewhere, such as the Han River. However, no empirical evidence exists to support this assumption yet. Second, the investigation of genetic admixture between clades A and B was not conducted in this study because the mitochondrial loci were only used. Perhaps, genotyping using nuclear loci is necessary through further studies.

*N. breviconcha* and *N. douglasiae* clade A, which have been found to inhabit only the Korean Peninsula, are believed to have very important values in elucidating the evolutionary and demographic history of *Nodularia* species in East Asia. It is quite sure that these species are very useful for tracking the structure and changes of past freshwater systems that have formed on the Korean Peninsula. Given that most studies regarding the historical changes of freshwater systems have been centered on fish, comparative studies using these freshwater mussel species would be very effective and provide new perspectives on it. Considering their ecological and phylogeographical importance, concrete and sustainable management plans for *N. breviconcha* and *N. douglasiae* clade A should be established to preserve their historical imprints on the Korean Peninsula in the near future.

## Methods

### Sample collection and DNA extraction

A total of 86 N*. douglasiae* and 83 N*. breviconcha* individuals were collected from the seven (Bukhan, Geum, Nakdong, Seomjin, Tamjin, Yeongsan, and Mangyeong) and six (Bukhan, Geum, Namhan, Seomjin, Tamjin, and Yeongsan) rivers in South Korea between 2016 and 2017, respectively (Fig. 1; Supplementary Fig. S1). The collected individuals were fixed in 95% alcohol in the field, and were taken to the laboratory. Species identification was performed based on the shell morphology. The genomic DNA was isolated from muscle tissues (foot) using a DNeasy Blood and Tissue Kit (QIAGEN, Valencia, California, USA) following the manufacturer’s protocol. The concentration of extracted DNA was evaluated using NanoDrop 2000 (Thermo Fisher Scientific Co, USA) and 1% agarose gel electrophoresis.

### PCR amplification and sequencing

To amplify partial mitochondrial DNA fragments of *CO1* and *16S rRNA*, and PCR was carried out using the previously known universal primers, LCO1490/HCO2198^29^ and LCO22me2/HCO700dy2^30^ for *CO1* and 16Sar-L-myt/16Sbr-H-myt^31^, 16SarL/16SbrH^32^, and 16Sar/16Sbr^33^ for *16S rRNA* (Supplementary Table S1). The information of the primers are shown in Supplementary Table S9. The thermal cycling profile consisted of a denaturation at 95 °C for 2 min, 35 cycles of 95 °C for 20 s, 48–50 °C for 40 s, and 72 °C for 1 min, and a final extension at 72 °C for 5 min. One microliter of each PCR product was electrophoresed on 1% agarose gel including eco-dye and observed under UV light. When the PCR bands were detected, the PCR products were purified using a QIAquick PCR Purification Kit (QIAGEN Co, USA) and directly sequenced with an ABI Prism 3730 DNA sequencer (PerkinElmer Inc, USA) using a Big Dye Termination Sequencing Kit (PerkinElmer Inc, USA). All the novel sequences of *CO1* and *16S rRNA* discovered in this study were deposited under the GenBank accession numbers (Supplementary Tables S5 and S6).

### Population genetic analyses

The nucleotide sequences of mitochondrial *CO1* and *16S rRNA* obtained from *N. douglasiae* and *N. breviconcha* were aligned using Clustal X2^34^ and BioEdit 7.2.5^35^. The identification of variable and parsimoniously informative sites and the number of haplotypes (*h*) were estimated using DnaSP 6.11^36^. A mismatch distribution analysis (MDA)^37^ was also conducted to infer demographic stability of phylogenetic clades and species using DnaSP 6.11. Based on the haplotype list generated from DnaSP, the number of private haplotypes unique to each population was determined (Supplementary Tables S1–S4, and S7, S8). The Arlequin 3.5 program^38^ was used to examine population demographic history and evolutionary neutrality of *N. douglasiae* and *N. breviconcha* based on the tests of Tajima’s *D*^39^ and Fu’s *F*s^40^. A haplotype network was constructed to estimate gene genealogies using the statistical parsimony approach at the population level using PopART^41^. To further evaluate and visualize the geographic genetic structure among the populations, a Principal Coordinates Analysis (PCoA) was conducted using the DARwin 6.0.9 program^42^. A Bayesian skyline plot (BSP) was computed in the BEAST 2.6.0 program^43, 44^ to examine the historical demographic fluctuation since the time of the most recent common ancestor. We used the HKY substitution model and mutation rates of 2.0 × 10^–8^ under a strict molecular clock used by Liu et al.^12^. Markov chain Monte Carlo was run for 5 million steps, with sampling every 1,000 generations, and the TRACER 1.5 program^45^ was used to construct the BSP^46^.

### Phylogenetic analyses

For phylogenetic analyses, a Bayesian topology was inferred under the GTR + I + G model using MrBayes 3.2.2^47^, and a maximum likelihood tree was reconstructed under the GTR + I + G model in IQtree online site (https://iqtree.cibiv.univie.ac.at) for the *CO1* and *16S rRNA* haplotype datasets. The 86 *CO1* and 83 *16S rRNA* nucleotide sequences newly obtained from *N. douglasiae* and *N. breviconcha* inhabiting the Korean Peninsula were employed for the phylogenetic analyses. As listed in Supplementary Tables S5 and S6, previously reported data were retrieved from the NCBI GenBank and added to our final nucleotide sequence alignment sets. The added sequences were the haplotypes of the samples obtained from South Korea^10^, China^4, 12^, Japan^10^, Russia^20^, and Vietnam^10^ for *CO1* of *N. douglasiae*, and from Japan (*N. nipponensis* and *N. biwae*) and South Korea (*N. breviconcha*)^48^ for *16S rRNA*. The outgroup species used were *Unio pictorum*, *Cuneopsis heudei*, *Cuneopsis rufescens*, *Cuneopsis pisciculus*, and *Schistodesmus lampreyanus* for the *CO1* dataset and *Unio pictorum* and *Cuneopsis heudei* for the *16S rRNA* dataset.

### Divergence time estimation

Divergence time estimation of the nodes on the *Nodularia* phylogeny was conducted in the BEAST 2.6.0. program^43, 44^ based on *CO1* sequences. The BEAST analysis was conducted based on fossil calibration points using a lognormal relaxed molecular clock algorithm^49^ under the calibrated-Yule model. A HKY model was applied with correlations for gamma distribution, and we designated priors for outgroup taxa using a “Monophyly” option of the BEAUti 2 program as (Parreysiinae, (*Nodularia*)). The fossil calibrations adapted to the analysis were estimated from the outgroup taxa^50^, since fossil records of the genus *Nodularia* have not known yet. The absolute age of outgroup taxa was referred in Bolotov et al.^50^. Posterior distributions of parameter were estimated using 1,000,000 MCMC generations with sampled every 1,000 generations. In the TreeAnnotator 2.6.0 program^51^, the initial 20% of generations were removed as burn-in, and resultant 1,001 trees were combined to a maximum clade credibility tree. The consensus tree was visualized in the FigTree 1.4.2 program^52^. To estimate the distribution of a hypothetical common ancestor, a Bayesian binary MCMC method (BBM)^53^, which is implemented in the RASP 3.2 program^54^, was adapted to the BEAST tree. When running the program, three possible distributed areas were coded for the ingroup taxa as follows: A) the distribution range of the clade A of *N. douglasiae* including Bukhan River and Nakdong River in South Korea; B) the distribution range of the clade B of *N. douglasiae* including the rest of rivers in South Korea except Bukhan River and Nakdong River, Japan, Vietnam, Hongze lake and Taihu lake in China, and Amur River and Ussury River in Russia; C) The distribution range of the clade C including Poyang lake, Duguan lake, Qinglan lake, Gan River, Liangzi lake, Dongting lake, and Xiannv lake in China, and Onon River in Russia.

## Supplementary information

Supplementary file 1
