# Supplementary material for: Molecular phylogenetic, population genetic and demographic studies of Nodularia douglasiae and Nodularia breviconcha based on CO1 and 16S rRNA
Source: Sci Rep. 2020 Oct 6;10:16572. doi: 10.1038/s41598-020-72015-5 (PMC7538972; doi:10.1038/s41598-020-72015-5)
Supplement: Supplementary file 1 [file 41598_2020_72015_MOESM1_ESM.docx]

**Molecular phylogenetic, population genetic and demographic studies of *Nodularia douglasiae* and *Nodularia breviconcha* based on *CO1* and *16S rRNA***

Eun Hwa Choi^1,2,#^, Gyeongmin Kim^1,3#^, Seung Hyun Cha^1,#^, Jun-Sang Lee^4^, Shi Hyun Ryu^5^, Ho Young Suk^6^, Young Sup Lee^3^, Su Youn Baek^1,2,*^, and Ui Wook Hwang^1,2,*^

*****To whom co-correspondence should be addressed Prof. Ui Wook Hwang (email: [*uwhwang1@gmail.com*](mailto:uwhwang1@gmail.com))

*Scientific Reports*

2020. 05. 18.

Supplementary Table S1. Distribution of 25 *CO1* haplotypes found in 86 individuals of *Nodularia douglasiae* collected from the seven freshwater systems in the Korean Peninsula.

| **Haplotype** | **Accession**  **No.** | **Freshwater systems** | | | | | | | **Total** |
| --- | --- | --- | --- | --- | --- | --- | --- | --- | --- |
|  |  | **BH** | **GM** | **MG** | **ND** | **SJ** | **YS** | **TJ** |  |
| DKCH01 | MN495497 | 11 | 4 |  | 2 |  |  |  | 17 |
| DKCH02 | MN495498 | 3 |  |  |  |  |  |  | 3 |
| DKCH03 | MN495499 | 2 |  |  |  |  |  |  | 2 |
| DKCH04 | MN495500 |  | 7 |  |  |  |  |  | 7 |
| DKCH05 | MN495501 |  | 1 |  |  |  |  |  | 1 |
| DKCH06 | MN495502 |  | 11 |  |  |  |  |  | 11 |
| DKCH07 | MN495503 |  | 1 |  | 8 |  |  |  | 9 |
| DKCH08 | MN495513 |  | 1 |  |  |  |  |  | 1 |
| DKCH09 | MN495514 |  | 1 |  |  |  |  |  | 1 |
| DKCH10 | MN495515 |  |  | 2 |  |  |  |  | 2 |
| DKCH11 | MN495516 |  |  | 1 |  |  |  |  | 1 |
| DKCH12 | MN495517 |  |  | 2 |  | 5 |  |  | 7 |
| DKCH13 | MN495504 |  |  |  | 2 |  |  |  | 2 |
| DKCH14 | MN495505 |  |  |  | 1 |  |  |  | 1 |
| DKCH15 | MN495506 |  |  |  | 7 |  |  |  | 7 |
| DKCH16 | MN495507 |  |  |  | 2 |  |  |  | 2 |
| DKCH17 | MN495508 |  |  |  | 2 |  |  |  | 2 |
| DKCH18 | MN495509 |  |  |  | 1 |  |  |  | 1 |
| DKCH19 | MN495510 |  |  |  | 1 |  |  |  | 1 |
| DKCH20 | MN495511 |  |  |  | 1 |  |  |  | 1 |
| DKCH21 | MN495512 |  |  |  | 1 |  |  |  | 1 |
| DKCH22 | MN495520 |  |  |  | 1 |  |  |  | 1 |
| DKCH23 | MN495521 |  |  |  | 1 |  |  |  | 1 |
| DKCH24 | MN495519 |  |  |  |  |  |  | 1 | 1 |
| DKCH25 | MN495518 |  |  |  |  |  | 3 |  | 1 |
| **Total** |  | **16** | **26** | **5** | **30** | **5** | **3** | **1** | **86** |

BH, Bukhan river; GM, Geum river; MG, Mangyeong river; ND, Nakdong river; SJ, Seomjin river; YS, Yeongsan river; TJ, Tamjin river

Supplementary Table S2. Distribution of 16 *CO1* haplotypes found in 83 individuals of *Nodularia breviconcha* collected from the six freshwater systems in the Korean Peninsula.

| **Haplotype** | **Accession**  **No.** | **Freshwater systems** | | | | | | **Total** |
| --- | --- | --- | --- | --- | --- | --- | --- | --- |
|  |  | **BH** | **GM** | **NH** | **SJ** | **YS** | **TJ** |  |
| SKCH01 | MN495522 | 12 |  | 28 |  |  |  | 40 |
| SKCH02 | MN495523 | 1 |  | 5 |  |  |  | 6 |
| SKCH03 | MN495524 | 1 |  |  |  |  |  | 1 |
| SKCH04 | MN495531 | 1 |  |  |  |  |  | 1 |
| SKCH05 | MN495525 | 11 |  | 1 |  |  |  | 12 |
| SKCH06 | MN495526 | 3 |  |  |  |  |  | 3 |
| SKCH07 | MN495532 |  | 1 |  |  |  |  | 1 |
| SKCH08 | MN495527 |  |  | 2 |  |  |  | 2 |
| SKCH09 | MN495528 |  |  | 3 |  |  |  | 3 |
| SKCH10 | MN495529 |  |  | 3 |  |  |  | 3 |
| SKCH11 | MN495530 |  |  | 1 |  |  |  | 1 |
| SKCH12 | MN495533 |  |  |  | 1 |  | 2 | 3 |
| SKCH13 | MN495534 |  |  |  | 1 |  |  | 1 |
| SKCH14 | MN495535 |  |  |  |  |  | 1 | 1 |
| SKCH15 | MN495536 |  |  |  |  | 4 |  | 4 |
| SKCH16 | MN495537 |  |  |  |  | 1 |  | 1 |
| **Total** |  | **29** | **1** | **43** | **2** | **5** | **3** | **83** |

BH, Bukhan river; GM, Geum river; NH, Namhan river; SJ, Seomjin river; YS, Yeongsan river; TJ, Tamjin river

Supplementary Table S3. Distribution of 16 *16S rRNA* haplotypes found in 79 individuals of *Nodularia douglasiae* collected from the six freshwater systems in the Korean Peninsula.

| **Haplotype** | **Accession**  **No.** | **Freshwater systems** | | | | | | **Total** |
| --- | --- | --- | --- | --- | --- | --- | --- | --- |
|  |  | **BH** | **GM** | **ND** | **SJ** | **YS** | **TJ** |  |
| DKSH01 | MN495472 | 10 |  |  |  |  |  | 10 |
| DKSH02 | MN495473 | 4 | 1 |  |  |  |  | 5 |
| DKSH03 | MN495474 | 2 | 3 | 3 |  | 3 |  | 11 |
| DKSH04 | MN495475 |  | 8 | 19 |  |  |  | 27 |
| DKSH05 | MN495476 |  | 9 |  |  |  |  | 9 |
| DKSH06 | MN495477 |  | 1 |  |  |  |  | 1 |
| DKSH07 | MN495478 |  | 1 |  |  |  |  | 1 |
| DKSH08 | MN495479 |  | 1 |  |  |  |  | 1 |
| DKSH09 | MN495480 |  | 1 |  |  |  |  | 1 |
| DKSH10 | MN495485 |  | 1 |  | 3 |  | 1 | 5 |
| DKSH11 | MN495481 |  |  | 3 |  |  |  | 3 |
| DKSH12 | MN495482 |  |  | 1 |  |  |  | 1 |
| DKSH13 | MN495483 |  |  | 1 |  |  |  | 1 |
| DKSH14 | MN495484 |  |  | 1 |  |  |  | 1 |
| DKSH15 | MN495486 |  |  | 1 |  |  |  | 1 |
| DKSH16 | MN495487 |  |  | 1 |  |  |  | 1 |
| **Total** |  | **16** | **26** | **30** | **3** | **3** | **1** | **79** |

BH, Bukhan river; GM, Geum river; ND, Nakdong river; SJ, Seomjin river; YS, Yeongsan river; TJ, Tamjin river

Supplementary Table S4. Distribution of 5 *16S rRNA* haplotypes found in 79 individuals of *Nodularia breviconcha* collected from the five freshwater systems in the Korean Peninsula.

| **Haplotype** | **Accession**  **No.** | **Freshwater systems** | | | | | **Total** |
| --- | --- | --- | --- | --- | --- | --- | --- |
|  |  | **BH** | **NH** | **SJ** | **YS** | **TJ** |  |
| SKSH01 | MN495488 | 26 | 41 |  | 4 | 2 | 73 |
| SKSH02 | MN495489 | 2 |  |  |  |  | 2 |
| SKSH03 | MN495491 | 1 |  | 1 |  |  | 2 |
| SKSH04 | MN495492 |  | 1 |  |  |  | 1 |
| SKSH05 | MN495493 |  | 1 |  |  |  | 1 |
| **Total** |  | **29** | **43** | **1** | **4** | **2** | **79** |

BH, Bukhan river; NH, Namhan river; SJ, Seomjin river; YS, Yeongsan river; TJ, Tamjin river

Supplementary Table S5. Summary of information of 197 *CO1* haplotypes from the six *Nodularia* species and 5 outgroups used in the present analyses.

| **No.** | **Species** | **Country** | **Location** | **Accession No.** | **Haplotype** | **Reference** |
| --- | --- | --- | --- | --- | --- | --- |
| **Ingroup** | | | | | | |
| 01 | *Nodularia douglasiae* | South Korea | Bukhan river | MN495497 | DKCH01 | Present study |
| 02 | *Nodularia douglasiae* | South Korea | Bukhan river | MN495498 | DKCH02 | Present study |
| 03 | *Nodularia douglasiae* | South Korea | Bukhan river | MN495499 | DKCH03 | Present study |
| 04 | *Nodularia douglasiae* | South Korea | Geum river | MN495500 | DKCH04 | Present study |
| 05 | *Nodularia douglasiae* | South Korea | Geum river | MN495501 | DKCH05 | Present study |
| 06 | *Nodularia douglasiae* | South Korea | Geum river | MN495502 | DKCH06 | Present study |
| 07 | *Nodularia douglasiae* | South Korea | Geum river | MN495503 | DKCH07 | Present study |
| 08 | *Nodularia douglasiae* | South Korea | Geum river | MN495513 | DKCH08 | Present study |
| 09 | *Nodularia douglasiae* | South Korea | Geum river | MN495514 | DKCH09 | Present study |
| 10 | *Nodularia douglasiae* | South Korea | Mangyeoung river | MN495515 | DKCH10 | Present study |
| 11 | *Nodularia douglasiae* | South Korea | Mangyeoung river | MN495516 | DKCH11 | Present study |
| 12 | *Nodularia douglasiae* | South Korea | Mangyeoung river | MN495517 | DKCH12 | Present study |
| 13 | *Nodularia douglasiae* | South Korea | Nakdong river | MN495504 | DKCH13 | Present study |
| 14 | *Nodularia douglasiae* | South Korea | Nakdong river | MN495505 | DKCH14 | Present study |
| 15 | *Nodularia douglasiae* | South Korea | Nakdong river | MN495506 | DKCH15 | Present study |
| 16 | *Nodularia douglasiae* | South Korea | Nakdong river | MN495507 | DKCH16 | Present study |
| 17 | *Nodularia douglasiae* | South Korea | Nakdong river | MN495508 | DKCH17 | Present study |
| 18 | *Nodularia douglasiae* | South Korea | Nakdong river | MN495509 | DKCH18 | Present study |
| 19 | *Nodularia douglasiae* | South Korea | Nakdong river | MN495510 | DKCH19 | Present study |
| 20 | *Nodularia douglasiae* | South Korea | Nakdong river | MN495511 | DKCH20 | Present study |
| 21 | *Nodularia douglasiae* | South Korea | Nakdong river | MN495512 | DKCH21 | Present study |
| 22 | *Nodularia douglasiae* | South Korea | Nakdong river | MN495518 | DKCH22 | Present study |
| 23 | *Nodularia douglasiae* | South Korea | Nakdong river | MN495519 | DKCH23 | Present study |
| 24 | *Nodularia douglasiae* | South Korea | Tamjin river | MN495520 | DKCH24 | Present study |
| 25 | *Nodularia douglasiae* | South Korea | Yeongsan river | MN495521 | DKCH25 | Present study |
| 26 | *Nodularia douglasiae* | South Korea | Geum river | MT020664 | DKCH08 | Lopes-Lima *et al*. 2020 |
| 27 | *Nodularia douglasiae* | South Korea | Yeongsan river | MT020665 | DKCH25 | Lopes-Lima *et al*. 2020 |
| 28 | *Nodularia douglasiae* | South Korea | Nakdong river | MT020668 | DKCH15 | Lopes-Lima *et al*. 2020 |
| 29 | *Nodularia douglasiae* | South Korea | Mangyeong river | MT020669 | DKCH10 | Lopes-Lima *et al*. 2020 |
| 30 | *Nodularia douglasiae* | China | Liangzi lake | MG210495 | DCCH25 | Liu *et al*. 2017 |
| 31 | *Nodularia douglasiae* | China | Liangzi lake | MG210496 | DCCH11 | Liu *et al*. 2017 |
| 32 | *Nodularia douglasiae* | China | Liangzi lake | MG210497 | DCCH24 | Liu *et al*. 2017 |
| 33 | *Nodularia douglasiae* | China | Liangzi lake | MG210498 | DCCH23 | Liu *et al*. 2017 |
| 34 | *Nodularia douglasiae* | China | Liangzi lake | MG210499 | DCCH05 | Liu *et al*. 2017 |
| 35 | *Nodularia douglasiae* | China | Liangzi lake | MG210500 | DCCH08 | Liu *et al*. 2017 |
| **No.** | **Species** | **Country** | **Location** | **Accession No.** | **Haplotype** | **Reference** |
| 36 | *Nodularia douglasiae* | China | Liangzi lake | MG210501 | DCCH11 | Liu *et al*. 2017 |
| 37 | *Nodularia douglasiae* | China | Liangzi lake | MG210502 | DCCH22 | Liu *et al*. 2017 |
| 38 | *Nodularia douglasiae* | China | Dongting lake | MG210503 | DCCH21 | Liu *et al*. 2017 |
| 39 | *Nodularia douglasiae* | China | Dongting lake | MG210504 | DCCH20 | Liu *et al*. 2017 |
| 40 | *Nodularia douglasiae* | China | Dongting lake | MG210505 | DCCH19 | Liu *et al*. 2017 |
| 41 | *Nodularia douglasiae* | China | Dongting lake | MG210506 | DCCH04 | Liu *et al*. 2017 |
| 42 | *Nodularia douglasiae* | China | Dongting lake | MG210507 | DCCH04 | Liu *et al*. 2017 |
| 43 | *Nodularia douglasiae* | China | Dongting lake | MG210508 | DCCH16 | Liu *et al*. 2017 |
| 44 | *Nodularia douglasiae* | China | Dongting lake | MG210509 | DCCH18 | Liu *et al*. 2017 |
| 45 | *Nodularia douglasiae* | China | Dongting lake | MG210510 | DCCH04 | Liu *et al*. 2017 |
| 46 | *Nodularia douglasiae* | China | Poyang lake | MG210511 | DCCH04 | Liu *et al*. 2017 |
| 47 | *Nodularia douglasiae* | China | Poyang lake | MG210514 | DCCH17 | Liu *et al*. 2017 |
| 48 | *Nodularia douglasiae* | China | Poyang lake | MG210515 | DCCH04 | Liu *et al*. 2017 |
| 49 | *Nodularia douglasiae* | China | Poyang lake | MG210518 | DCCH17 | Liu *et al*. 2017 |
| 50 | *Nodularia douglasiae* | China | Gan river | MG210519 | DCCH04 | Liu *et al*. 2017 |
| 51 | *Nodularia douglasiae* | China | Gan river | MG210520 | DCCH08 | Liu *et al*. 2017 |
| 52 | *Nodularia douglasiae* | China | Gan river | MG210521 | DCCH15 | Liu *et al*. 2017 |
| 53 | *Nodularia douglasiae* | China | Gan river | MG210522 | DCCH16 | Liu *et al*. 2017 |
| 54 | *Nodularia douglasiae* | China | Gan river | MG210523 | DCCH05 | Liu *et al*. 2017 |
| 55 | *Nodularia douglasiae* | China | Gan river | MG210524 | DCCH15 | Liu *et al*. 2017 |
| 56 | *Nodularia douglasiae* | China | Gan river | MG210525 | DCCH14 | Liu *et al*. 2017 |
| 57 | *Nodularia douglasiae* | China | Gan river | MG210526 | DCCH09 | Liu *et al*. 2017 |
| 58 | *Nodularia douglasiae* | China | Gan river | MG210528 | DCCH04 | Liu *et al*. 2017 |
| 59 | *Nodularia douglasiae* | China | Gan river | MG210529 | DCCH13 | Liu *et al*. 2017 |
| 60 | *Nodularia douglasiae* | China | Gan river | MG210530 | DCCH12 | Liu *et al*. 2017 |
| 61 | *Nodularia douglasiae* | China | Gan river | MG210531 | DCCH11 | Liu *et al*. 2017 |
| 62 | *Nodularia douglasiae* | China | Gan river | MG210533 | DCCH10 | Liu *et al*. 2017 |
| 63 | *Nodularia douglasiae* | China | Gan river | MG210534 | DCCH08 | Liu *et al*. 2017 |
| 64 | *Nodularia douglasiae* | China | Xiannv lake | MG210537 | DCCH05 | Liu *et al*. 2017 |
| 65 | *Nodularia douglasiae* | China | Xiannv lake | MG210538 | DCCH06 | Liu *et al*. 2017 |
| 66 | *Nodularia douglasiae* | China | Xiannv lake | MG210540 | DCCH09 | Liu *et al*. 2017 |
| 67 | *Nodularia douglasiae* | China | Xiannv lake | MG210542 | DCCH06 | Liu *et al*. 2017 |
| 68 | *Nodularia douglasiae* | China | Hongze lake | MG210543 | DCCH06 | Liu *et al*. 2017 |
| 69 | *Nodularia douglasiae* | China | Hongze lake | MG210544 | DCCH08 | Liu *et al*. 2017 |
| 70 | *Nodularia douglasiae* | China | Hongze lake | MG210546 | DCCH02  /DJCH01 | Liu *et al*. 2017 |
| 71 | *Nodularia douglasiae* | China | Hongze lake | MG210547 | DCCH07 | Liu *et al*. 2017 |
| 72 | *Nodularia douglasiae* | China | Hongze lake | MG210549 | DCCH06 | Liu *et al*. 2017 |
| 73 | *Nodularia douglasiae* | China | Hongze lake | MG210550 | DCCH05 | Liu *et al*. 2017 |
| **No.** | **Species** | **Country** | **Location** | **Accession No.** | **Haplotype** | **Reference** |
| 74 | *Nodularia douglasiae* | China | Taihu lake | MG210551 | DCCH04 | Liu *et al*. 2017 |
| 75 | *Nodularia douglasiae* | China | Taihu lake | MG210552 | DCCH03 | Liu *et al*. 2017 |
| 76 | *Nodularia douglasiae* | China | Taihu lake | MG210553 | DCCH26 | Liu *et al*. 2017 |
| 77 | *Nodularia douglasiae* | China | Taihu lake | MG210554 | DCCH16 | Liu *et al*. 2017 |
| 78 | *Nodularia douglasiae* | China | Taihu lake | MG210555 | DCCH03 | Liu *et al*. 2017 |
| 79 | *Nodularia douglasiae* | China | Taihu lake | MG210556 | DCCH14 | Liu *et al*. 2017 |
| 80 | *Nodularia douglasiae* | China | Taihu lake | MG210557 | DCCH26 | Liu *et al*. 2017 |
| 81 | *Nodularia douglasiae* | China | Taihu lake | MG210558 | DCCH09 | Liu *et al*. 2017 |
| 82 | *Nodularia douglasiae* | China | Yangtze basin | KJ434521 | DCCH27 | Ouyang *et al*. 2015 |
| 83 | *Nodularia douglasiae* | China | Yangtze basin | KJ434522 | DCCH05 | Ouyang *et al.* 2015 |
| 84 | *Nodularia douglasiae* | China | Yangtze river | MF975690 | DCCH01 | GenBank |
| 85 | *Nodularia douglasiae* | China | Poyang lake basin | MH822323 | DCCH16 | Liu *et al*. 2019 |
| 86 | *Nodularia douglasiae* | China | Poyang lake basin | MH822324 | DCCH15 | Liu *et al*. 2019 |
| 87 | *Nodularia douglasiae* | China | Poyang lake basin | MH822325 | DCCH06 | Liu *et al*. 2019 |
| 88 | *Nodularia douglasiae* | China | Poyang lake basin | MH822328 | DCCH46 | Liu *et al*. 2019 |
| 89 | *Nodularia douglasiae* | China | Poyang lake basin | MH822331 | DCCH04 | Liu *et al*. 2019 |
| 90 | *Nodularia douglasiae* | China | Poyang lake basin | MH822332 | DCCH08 | Liu *et al*. 2019 |
| 91 | *Nodularia douglasiae* | China | Poyang lake basin | MH822333 | DCCH05 | Liu *et al*. 2019 |
| 92 | *Nodularia douglasiae* | China | Poyang lake basin | MH822334 | DCCH14 | Liu *et al*. 2019 |
| 93 | *Nodularia douglasiae* | China | Poyang lake basin | MH822335 | DCCH09 | Liu *et al*. 2019 |
| 94 | *Nodularia douglasiae* | China | Poyang lake basin | MH822336 | DCCH04 | Liu *et al*. 2019 |
| 95 | *Nodularia douglasiae* | China | Poyang lake basin | MH822337 | DCCH13 | Liu *et al*. 2019 |
| 96 | *Nodularia douglasiae* | China | Poyang lake basin | MH822338 | DCCH12 | Liu *et al*. 2019 |
| 97 | *Nodularia douglasiae* | China | Poyang lake basin | MH822339 | DCCH11 | Liu *et al*. 2019 |
| 98 | *Nodularia douglasiae* | China | Poyang lake basin | MH822340 | DCCH10 | Liu *et al*. 2019 |
| 99 | *Nodularia douglasiae* | China | Poyang lake basin | MH822341 | DCCH17 | Liu *et al*. 2019 |
| 100 | *Nodularia douglasiae* | China | Poyang lake basin | MH822342 | DCCH27 | Liu *et al*. 2019 |
| 101 | *Nodularia douglasiae* | China | Poyang lake basin | MH822343 | DCCH31 | Liu *et al*. 2019 |
| 102 | *Nodularia douglasiae* | China | Poyang lake basin | MH822345 | DCCH43 | Liu *et al*. 2019 |
| 103 | *Nodularia douglasiae* | China | Poyang lake basin | MH822348 | DCCH11 | Liu *et al*. 2019 |
| 104 | *Nodularia douglasiae* | China | Poyang lake basin | MH822351 | DCCH02  /DJCH01 | Liu *et al*. 2019 |
| 105 | *Nodularia douglasiae* | China | Poyang lake basin | MH822352 | DCCH38 | Liu *et al*. 2019 |
| 106 | *Nodularia douglasiae* | China | Poyang lake basin | MH822353 | DCCH37 | Liu *et al*. 2019 |
| 107 | *Nodularia douglasiae* | China | Poyang lake basin | MH822355 | DCCH36 | Liu *et al*. 2019 |
| 108 | *Nodularia douglasiae* | China | Poyang lake basin | MH822356 | DCCH35 | Liu *et al*. 2019 |
| 109 | *Nodularia douglasiae* | China | Poyang lake basin | MH822359 | DCCH07 | Liu *et al*. 2019 |
| 110 | *Nodularia douglasiae* | China | Poyang lake basin | MH822363 | DCCH27 | Liu *et al*. 2019 |
| 111 | *Nodularia douglasiae* | China | Poyang lake basin | MH822364 | DKCH21 | Liu *et al*. 2019 |
| **No.** | **Species** | **Country** | **Location** | **Accession No.** | **Haplotype** | **Reference** |
| 112 | *Nodularia douglasiae* | China | Duguan lake | MG463024 | DCCH09 | Huang *et al*. 2018 |
| 113 | *Nodularia douglasiae* | China | Duguan lake | MG463025 | DCCH14 | Huang *et al*. 2018 |
| 114 | *Nodularia douglasiae* | China | Gan river | MG463026 | DCCH31 | Huang *et al*. 2018 |
| 115 | *Nodularia douglasiae* | China | Gan river | MG463027 | DCCH48 | Huang *et al*. 2018 |
| 116 | *Nodularia douglasiae* | China | Gan river | MG463028 | DCCH27 | Huang *et al*. 2018 |
| 117 | *Nodularia douglasiae* | China | Qinglan lake | MG463030 | DCCH05 | Huang *et al*. 2018 |
| 118 | *Nodularia douglasiae* | China | Qinglan lake | MG463031 | DCCH07 | Huang *et al*. 2018 |
| 119 | *Nodularia douglasiae* | China | Qinglan lake | MG463032 | DCCH13 | Huang *et al*. 2018 |
| 120 | *Nodularia douglasiae* | China | Gan river | MG463033 | DCCH05 | Huang *et al*. 2018 |
| 121 | *Nodularia douglasiae* | China | Yantze basin | KJ434520 | DCCH31 | Uyang *et al.* unp. |
| 122 | *Nodularia douglasiae* | Japan | Fukuro river | MT020676 | DKCH21 | Lopes-Lima  *et al*. 2020 |
| 123 | *Nodularia douglasiae* | Japan | Biwa lake | MT020672 | DCCH02/  DJCH01 | Lopes-Lima  *et al*. 2020 |
| 124 | *Nodularia douglasiae* | Japan | Okayama | MF975688 | DJCH02 | Klishko *et al.* 2018 |
| 125 | *Nodularia douglasiae* | Japan | Okayama | MF975689 | DCCH02/DJCH01 | Klishko *et al*. 2018 |
| 126 | *Nodularia douglasiae* | Russia | Amur river | MF975692 | DRCH02 | Klishko *et al*. 2018 |
| 127 | *Nodularia douglasiae* | Russia | Onon River | MF975693 | DRCH05 | Klishko *et al*. 2018 |
| 128 | *Nodularia douglasiae* | Russia | Onon River | MF975694 | DRCH04 | Klishko *et al*. 2018 |
| 129 | *Nodularia douglasiae* | Russia | Amur river | MF975695 | DRCH03 | Klishko *et al*. 2018 |
| 130 | *Nodularia douglasiae* | Russia | Amur river | MF975696 | DRCH02 | Klishko *et al*. 2018 |
| 131 | *Nodularia douglasiae* | Russia | Ussury river | MF975697 | DRCH01 | Klishko *et al*. 2018 |
| 132 | *Nodularia douglasiae* | Russia | Ussury river | MF975698 | DRCH01 | Klishko *et al*. 2018 |
| 133 | *Nodularia douglasiae* | Vietnam | Hanoi | MH248376 | DVCH01 | Lopes-Lima  *et al*. 2020 |
| 134 | *Nodularia* sp.1 | China | Hongze river | MG210545 | DCCH50 | Liu *et al*. 2017 |
| 135 | *Nodularia* sp.1 | China | Hongze river | MG210548 | DCCH50 | Liu *et al*. 2017 |
| 136 | *Nodularia* sp.2 | China | Poyang lake | MG210512 | DCCH30 | Liu *et al*. 2017 |
| 137 | *Nodularia* sp.2 | China | Poyang lake | MG210513 | DCCH28 | Liu *et al*. 2017 |
| 138 | *Nodularia* sp.2 | China | Poyang lake | MG210516 | DCCH30 | Liu *et al*. 2017 |
| 139 | *Nodularia* sp.2 | China | Poyang lake | MG210517 | DCCH28 | Liu *et al*. 2017 |
| 140 | *Nodularia* sp.2 | China | Gan river | MG210527 | DCCH28 | Liu *et al*. 2017 |
| 141 | *Nodularia* sp.2 | China | Gan river | MG210532 | DCCH28 | Liu *et al*. 2017 |
| 142 | *Nodularia* sp.2 | China | Xiannv lake | MG210535 | DCCH28 | Liu *et al*. 2017 |
| 143 | *Nodularia* sp.2 | China | Xiannv lake | MG210536 | DCCH30 | Liu *et al*. 2017 |
| 144 | *Nodularia* sp.2 | China | Xiannv lake | MG210539 | DCCH29 | Liu *et al*. 2017 |
| 145 | *Nodularia* sp.2 | China | Xiannv lake | MG210541 | DCCH28 | Liu *et al*. 2017 |
| 146 | *Nodularia* sp.2 | China | Poyang lake basin | MH822322 | DCCH28 | Liu *et al*. 2019 |
| 147 | *Nodularia* sp.2 | China | Poyang lake basin | MH822326 | DCCH30 | Liu *et al*. 2019 |
| 148 | *Nodularia* sp.2 | China | Poyang lake basin | MH822327 | DCCH47 | Liu *et al*. 2019 |
| **No.** | **Species** | **Country** | **Location** | **Accession No.** | **Haplotype** | **Reference** |
| 149 | *Nodularia* sp.2 | China | Poyang lake basin | MH822329 | DCCH45 | Liu *et al*. 2019 |
| 150 | *Nodularia* sp.2 | China | Poyang lake basin | MH822330 | DCCH44 | Liu *et al*. 2019 |
| 151 | *Nodularia* sp.2 | China | Poyang lake basin | MH822346 | DCCH42 | Liu *et al*. 2019 |
| 152 | *Nodularia* sp.2 | China | Poyang lake basin | MH822347 | DCCH41 | Liu *et al*. 2019 |
| 153 | *Nodularia* sp.2 | China | Poyang lake basin | MH822349 | DCCH40 | Liu *et al*. 2019 |
| 154 | *Nodularia* sp.2 | China | Poyang lake basin | MH822350 | DCCH39 | Liu *et al*. 2019 |
| 155 | *Nodularia* sp.2 | China | Poyang lake basin | MH822357 | DCCH34 | Liu *et al*. 2019 |
| 156 | *Nodularia* sp.2 | China | Poyang lake basin | MH822358 | DCCH29 | Liu *et al*. 2019 |
| 157 | *Nodularia* sp.2 | China | Poyang lake basin | MH822360 | DCCH33 | Liu *et al*. 2019 |
| 158 | *Nodularia* sp.2 | China | Poyang lake basin | MH822361 | DCCH32 | Liu *et al*. 2019 |
| 159 | *Nodularia* sp.2 | China | Poyang lake basin | MH822362 | DCCH30 | Liu *et al*. 2019 |
| 160 | *Nodularia* sp.2 | China | Gan river | MG463023 | DCCH49 | Huang *et al*. 2018 |
| 161 | *Nodularia* sp.2 | China | Qinglan lake | MG463029 | DCCH28 | Huang *et al*. 2018 |
| 162 | *Nodularia nipponensis* | Japan | Abukuma river | MF975691 | NJCH01 | Klishko *et al*. 2018 |
| 163 | *Nodularia nipponensis* | Japan | Abukuma river | MT020673 | NJCH01 | Lopes-Lima *et al.* 2020 |
| 164 | *Nodularia nipponensis* | Japan | Kasumigaura lake | LC496352* | NJCH01 | Fukata & Masayuki, 2019 |
| 165 | *Nodularia nipponensis* | Japan | Biwa lake | MT020674 | NJCH02 | Lopes-Lima *et al.* 2020 |
| 166 | *Nodularia nipponensis* | Japan | Izunuma lake | MT020677 | NJCH03 | Lopes-Lima *et al.* 2020 |
| 167 | *Nodularia nipponensis* | Japan | Name river | MT020678 | NJCH04 | Lopes-Lima *et al.* 2020 |
| 168 | *Nodularia nipponensis* | Japan | Name river | MT020679 | NJCH05 | Lopes-Lima *et al.* 2020 |
| 169 | *Nodularia nipponensis* | Japan | Biwa lake | MT020680 | NJCH06 | Lopes-Lima *et al.* 2020 |
| 170 | *Nodularia nipponensis* | Japan | Biwa lake | MT020681 | NJCH07 | Lopes-Lima *et al.* 2020 |
| 171 | *Nodularia nuxpersicae* | Vietnam | Tây Bắc | KX822654 | *N. nuxpersicae* | Lopes-Lima *et al.* 2020 |
| 172 | *Nodularia breviconcha* | South Korea | Bukhan river | MN495522 | SKCH01 | Present study |
| 173 | *Nodularia breviconcha* | South Korea | Namhan river | MN495523 | SKCH02 | Present study |
| 174 | *Nodularia breviconcha* | South Korea | Bukhan river | MN495524 | SKCH03 | Present study |
| 175 | *Nodularia breviconcha* | South Korea | Bukhan river | MN495531 | SKCH04 | Present study |
| 176 | *Nodularia breviconcha* | South Korea | Bukhan river | MN495525 | SKCH05 | Present study |
| 177 | *Nodularia breviconcha* | South Korea | Bukhan river | MN495526 | SKCH06 | Present study |
| 178 | *Nodularia breviconcha* | South Korea | Geum river | MN495532 | SKCH07 | Present study |
| 179 | *Nodularia breviconcha* | South Korea | Namhan river | MN495527 | SKCH08 | Present study |
| 180 | *Nodularia breviconcha* | South Korea | Namhan river | MN495528 | SKCH09 | Present study |
| 181 | *Nodularia breviconcha* | South Korea | Namhan river | MN495529 | SKCH10 | Present study |
| 182 | *Nodularia breviconcha* | South Korea | Namhan river | MN495530 | SKCH11 | Present study |
| 183 | *Nodularia breviconcha* | South Korea | Seomjin river | MN495533 | SKCH12 | Present study |
| 184 | *Nodularia breviconcha* | South Korea | Seomjin river | MN495534 | SKCH13 | Present study |
| 185 | *Nodularia breviconcha* | South Korea | Tamjin river | MN495535 | SKCH14 | Present study |
| **No.** | **Species** | **Country** | **Location** | **Accession No.** | **Haplotype** | **Reference** |
| 186 | *Nodularia breviconcha* | South Korea | Yeongsan river | MN495536 | SKCH15 | Present study |
| 187 | *Nodularia breviconcha* | South Korea | Yeongsan river | MN495537 | SKCH16 | Present study |
| 188 | *Nodularia breviconcha* | South Korea | Seomjin river | MT020662 | SKCH12 | Lopes-Lima *et al.* 2020 |
| 189 | *Nodularia breviconcha* | South Korea | Han river | MT020663 | SKCH01 | Lopes-Lima *et al.* 2020 |
| 190 | *Nodularia breviconcha* | South Korea | Tamjin river | MT020666 | SKCH12 | Lopes-Lima *et al.* 2020 |
| 191 | *Nodularia breviconcha* | South Korea | Hyeonsan Stream | MT020667 | SKCH15 | Lopes-Lima *et al.* 2020 |
| 192 | *Nodularia breviconcha* | South Korea | Ungcheon Stream | MT020670 | SKCH07 | Lopes-Lima *et al.* 2020 |
| **Outgroup** | | | | | | |
| 193 | *Unio pictorum* | Poland | ­­— | NC015310 | *Unio pictorum* | Soroka & Burzynski. 2010 |
| 194 | *Cuneopsis heudei* | China | — | MF687348 | *Cuneopsis heudei* | Wang *et al*. 2018 |
| 195 | *Cuneopsis rufescens* | China | — | KX822640 | *Cuneopsis rufescens* | Lopes-Lima *et a*l. 2017 |
| 196 | *Cuneopsis pisciculus* | China | — | NC026306 | *Cuneopsis pisciculus* | Han *et al*. 2016 |
| 197 | *Schistodesmus lampreyanus* | China | — | KX822665 | *Schistodesmus lampreyanus* | Lopes-Lima *et al*. 2017 |

* Although it was originally reported as a member of *Nodularia douglasiae*, the present study revealed it to be a member of *Nodularia nipponensis*.

Supplementary Table S6. Summary of information of 33 *16S rRNA* haplotypes from the three *Nodularia* species and 2 outgroups used in the present analyses.

| **No.** | **Species** | **Country** | | **Location** | | **Accession No.** | | **Haplotype** | | **Reference** |
| --- | --- | --- | --- | --- | --- | --- | --- | --- | --- | --- |
| **Ingroup** | | | | | | | | | | |
| 01 | *Nodularia douglasiae* | South Korea | | Bukhan river | | MN495472 | | DKSH01 | | Present study |
| 02 | *Nodularia douglasiae* | South Korea | | Bukhan river | | MN495473 | | DKSH02 | | Present study |
| 03 | *Nodularia douglasiae* | South Korea | | Bukhan river | | MN495474 | | DKSH03 | | Present study |
| 04 | *Nodularia douglasiae* | South Korea | | Geum river | | MN495475 | | DKSH04 | | Present study |
| 05 | *Nodularia douglasiae* | South Korea | | Geum river | | MN495476 | | DKSH05 | | Present study |
| 06 | *Nodularia douglasiae* | South Korea | | Geum river | | MN495477 | | DKSH06 | | Present study |
| 07 | *Nodularia douglasiae* | South Korea | | Geum river | | MN495478 | | DKSH07 | | Present study |
| 08 | *Nodularia douglasiae* | South Korea | | Geum river | | MN495479 | | DKSH08 | | Present study |
| 09 | *Nodularia douglasiae* | South Korea | | Geum river | | MN495480 | | DKSH09 | | Present study |
| 10 | *Nodularia douglasiae* | South Korea | | Seomjin river | | MN495485 | | DKSH10 | | Present study |
| 11 | *Nodularia douglasiae* | South Korea | | Nakdong river | | MN495481 | | DKSH11 | | Present study |
| 12 | *Nodularia douglasiae* | South Korea | | Nakdong river | | MN495482 | | DKSH12 | | Present study |
| 13 | *Nodularia douglasiae* | South Korea | | Nakdong river | | MN495483 | | DKSH13 | | Present study |
| 14 | *Nodularia douglasiae* | South Korea | | Nakdong river | | MN495484 | | DKSH14 | | Present study |
| 15 | *Nodularia douglasiae* | South Korea | | Nakdong river | | MN495486 | | DKSH15 | | Present study |
| 16 | *Nodularia douglasiae* | South Korea | | Nakdong river | | MN495487 | | DKSH16 | | Present study |
| 17 | *Nodularia douglasiae* | China | | Yangtze | | NC026111 | | DCSH01 | | Xue *et al*. unp. |
| 18 | *Nodularia douglasiae* | Japan | | Wakayama | | LC223975 | | DJSH01 | | Sano *et al*. 2017 |
| 19 | *Nodularia douglasiae* | Japan | | Wakayama | | LC223977 | | DJSH02 | | Sano *et al*. 2017 |
| 20 | *Nodularia douglasiae* | Japan | | Nakama | | LC223978 | | DJSH03 | | Sano *et al*. 2017 |
| 21 | *Nodularia nipponensis* | Japan | | Kasumigaura lake | | LC496352* | | LC496352 | | Fukata & Masayuki, 2019 |
| 22 | *Nodularia nipponensis* | Japan | | Biwa | | LC223961 | | LC223961 | | Sano *et al*. 2017 |
| 23 | *Nodularia nipponensis* | Japan | | Biwa | | LC223962 | | LC223962 | | Sano *et al*. 2017 |
| 24 | *Nodularia nipponensis* | Japan | | Yamanashi | | LC223963 | | LC223963 | | Sano *et al*. 2017 |
| 25 | *Nodularia nipponensis* | Japan | | Yamanashi | | LC223964 | | LC223964 | | Sano *et al.* 2017 |
| 26 | *Nodularia nipponensis* | Japan | | Yamanashi | | LC223965 | | LC223965 | | Sano *et al*. 2017 |
| 27 | *Nodularia nipponensis* | Japan | | Nakama | | LC223976 | | LC223976 | | Sano et al. 2017 |
| 28 | *Nodularia breviconcha* | South Korea | | Bukhan river | | MN495488 | | SKSH01 | | Present study |
| 29 | *Nodularia breviconcha* | South Korea | | Bukhan river | | MN495489 | | SKSH02 | | Present study |
| 30 | *Nodularia breviconcha* | South Korea | | Bukhan river | | MN495491 | | SKSH03 | | Present study |
| 31 | *Nodularia breviconcha* | South Korea | | Namhan river | | MN495492 | | SKSH04 | | Present study |
| 32 | *Nodularia breviconcha* | South Korea | | Namhan river | | MN495493 | | SKSH05 | | Present study |
| **No.** | **Species** | **Country** | **Location** | | **Accession No.** | | **Haplotype** | | **Reference** | |
| **Outgroup** | | | | | | | | | | |
| 32 | *Unio pictorum* | Poland | — | | NC015310 | | *Unio pictorum* | | Soroka & Burzynski. 2010 | |
| 33 | *Cuneopsis heudei* | China | — | | MF687348 | | *Cuneopsis heudei* | | Wang *et al*. 2018 | |

* Although it was originally reported as a member of *Nodularia douglasiae*, the present study revealed it to be a member of *Nodularia nipponensis*.

Supplementary Table S7. The number of *Nodularia douglasiae* individuals along the 67 *CO1* haplotypes found from the five East Asian countries.

| **No.** | **Haplotype** | **Country** | | | | | **Total** |
| --- | --- | --- | --- | --- | --- | --- | --- |
|  |  | **South Korea** | **China** | **Russia** | **Japan** | **Vietnam** |  |
| 01 | DKCH01 | 17 |  |  |  |  | 17 |
| 02 | DKCH02 | 3 |  |  |  |  | 3 |
| 03 | DKCH03 | 2 |  |  |  |  | 2 |
| 04 | DKCH04 | 7 |  |  |  |  | 7 |
| 05 | DKCH05 | 1 |  |  |  |  | 1 |
| 06 | DKCH06 | 11 |  |  |  |  | 11 |
| 07 | DKCH07 | 9 |  |  |  |  | 9 |
| 08 | DKCH08 | 2(1)* |  |  |  |  | 2 |
| 09 | DKCH09 | 1 |  |  |  |  | 1 |
| 10 | DKCH10 | 3(1) * |  |  |  |  | 3 |
| 11 | DKCH11 | 1 |  |  |  |  | 1 |
| 12 | DKCH12 | 7 | 1 |  | 1 |  | 9 |
| 13 | DKCH13 | 2 |  |  |  |  | 2 |
| 14 | DKCH14 | 1 |  |  |  |  | 1 |
| 15 | DKCH15 | 8(1) * |  |  |  |  | 8 |
| 16 | DKCH16 | 2 |  |  |  |  | 2 |
| 17 | DKCH17 | 2 |  |  |  |  | 2 |
| 18 | DKCH18 | 1 |  |  |  |  | 1 |
| 19 | DKCH19 | 1 |  |  |  |  | 1 |
| 20 | DKCH20 | 1 |  |  |  |  | 1 |
| 21 | DKCH21 | 1 |  |  |  |  | 1 |
| 22 | DKCH22 | 1 |  |  |  |  | 1 |
| 23 | DKCH23 | 1 |  |  |  |  | 1 |
| 24 | DKCH24 | 1 |  |  |  |  | 1 |
| 25 | DKCH25 | 4(1) * |  |  |  |  | 4 |
| 26 | DRCH01 |  |  | 2 |  |  | 2 |
| 27 | DRCH02 |  |  | 2 |  |  | 2 |
| 28 | DRCH03 |  |  | 1 |  |  | 1 |
| 29 | DRCH04 |  |  | 1 |  |  | 1 |
| 30 | DRCH05 |  |  | 1 |  |  | 1 |
| 31 | DCCH01 |  | 1 |  |  |  | 1 |
| 32 | DCCH02/DJCH01 |  | 2 |  | 2 |  | 4 |
| 33 | DCCH03 |  | 2 |  |  |  | 2 |
| 34 | DCCH04 |  | 10 |  |  |  | 10 |
| 35 | DCCH05 |  | 8 |  |  |  | 8 |
| 36 | DCCH06 |  | 5 |  |  |  | 5 |
| 37 | DCCH07 |  | 3 |  |  |  | 3 |
| 38 | DCCH08 |  | 5 |  |  |  | 5 |
| 39 | DCCH09 |  | 5 |  |  |  | 5 |
| 40 | DCCH10 |  | 2 |  |  |  | 2 |
| 41 | DCCH11 |  | 5 |  |  |  | 5 |
| 42 | DCCH12 |  | 2 |  |  |  | 2 |
| 43 | DCCH13 |  | 3 |  |  |  | 3 |
| **No.** | **Haplotype** | **Country** | | | | | **Total** |
|  |  | **South Korea** | **China** | **Russia** | **Japan** | **Vietnam** |  |
| 44 | DCCH14 |  | 4 |  |  |  | 4 |
| 45 | DCCH15 |  | 3 |  |  |  | 3 |
| 46 | DCCH16 |  | 4 |  |  |  | 4 |
| 47 | DCCH17 |  | 3 |  |  |  | 3 |
| 48 | DCCH18 |  | 1 |  |  |  | 1 |
| 49 | DCCH19 |  | 1 |  |  |  | 1 |
| 50 | DCCH20 |  | 1 |  |  |  | 1 |
| 51 | DCCH21 |  | 1 |  |  |  | 1 |
| 52 | DCCH22 |  | 1 |  |  |  | 1 |
| 53 | DCCH23 |  | 1 |  |  |  | 1 |
| 54 | DCCH24 |  | 1 |  |  |  | 1 |
| 55 | DCCH25 |  | 1 |  |  |  | 1 |
| 56 | DCCH26 |  | 2 |  |  |  | 2 |
| 57 | DCCH27 |  | 4 |  |  |  | 4 |
| 58 | DCCH31 |  | 3 |  |  |  | 3 |
| 59 | DCCH35 |  | 1 |  |  |  | 1 |
| 60 | DCCH36 |  | 1 |  |  |  | 1 |
| 61 | DCCH37 |  | 1 |  |  |  | 1 |
| 62 | DCCH38 |  | 1 |  |  |  | 1 |
| 63 | DCCH43 |  | 1 |  |  |  | 1 |
| 64 | DCCH46 |  | 1 |  |  |  | 1 |
| 65 | DCCH48 |  | 1 |  |  |  | 1 |
| 66 | DJCH02 |  |  |  | 1 |  | 1 |
| 67 | DVCH01 |  |  |  |  | 1 | 1 |
| **Total** | | **90** | **92** | **7** | **4** | **1** | **194** |

* indicates the number of haplotypes reported by Lopes-Lima *et al*. (2020) in the Korean Peninsula

Supplementary Table S8. The number of *Nodularia douglasiae* individuals along the 20 *16S rRNA* haplotypes found from three East Asian countries.

| **No.** | **Haplotype** | **Country** | | | **Total** |
| --- | --- | --- | --- | --- | --- |
|  |  | **South Korea** | **China** | **Japan** |  |
| 01 | DKSH01 | 10 |  |  | 10 |
| 02 | DKSH02 | 5 |  |  | 5 |
| 03 | DKSH03 | 11 |  |  | 11 |
| 04 | DKSH04 | 27 |  |  | 27 |
| 05 | DKSH05 | 9 |  |  | 9 |
| 06 | DKSH06 | 1 |  |  | 1 |
| 07 | DKSH07 | 1 |  |  | 1 |
| 08 | DKSH08 | 1 |  |  | 1 |
| 09 | DKSH09 | 1 |  |  | 1 |
| 10 | DKSH10 | 5 |  |  | 5 |
| 11 | DKSH11 | 3 |  |  | 3 |
| 12 | DKSH12 | 1 |  |  | 1 |
| 13 | DKSH13 | 1 |  |  | 1 |
| 14 | DKSH14 | 1 |  |  | 1 |
| 15 | DKSH15 | 1 |  |  | 1 |
| 16 | DKSH16 | 1 |  |  | 1 |
| 17 | DCSH01 |  | 1 |  | 1 |
| 18 | DJSH01 |  |  | 1 | 1 |
| 19 | DJSH02 |  |  | 1 | 1 |
| 20 | DJSH03 |  |  | 1 | 1 |
| **Total** | | **79** | **1** | **3** | **83** |

Supplementary Table S9. The information of primers used for PCR amplifications of *CO1* and *16S rRNA.*

| **Gene** | **Primer** | **Sequence (5´→3´)** |
| --- | --- | --- |
| ***CO1*** | LCO1490^1)^ | GGT CAA CAA ATC ATA AAG ATA TTG G |
|  | HCO2198^1)^ | TAA ACT TCA GGG TGA CCA AAA AAT CA |
|  | LCO22me2^2)^ | GGT CAA CAA AYC ATA ARG ATA TTG G |
|  | HCO700dy2^2)^ | TCA GGG TGA CCA AAA AAY CA |
|  |  |  |
| ***16S rRNA*** | 16Sar-L-myt^3)^ | CGA CTG TTT AAC AAA AAC AT |
|  | 16Sbr-H-myt^3)^ | CCG TTC TGA ACT CAG CTC ATG T |
|  | 16Sar-L^4)^ | CGC CTG TTT ATC AAA AAC AT |
|  | 16Sbr-H^4)^ | CCG GTC TGA ACT CAG ATC ACG T |
|  | 16Sar^5)^ | CGC CTG TTT AAC AAA AAC AT |
|  | 16Sbr^5)^ | CCG GTT TGA ACT CAG ATC ATG T |
| ^1)^Folmer *et al*. (1994), ^2)^Walker *et al*. (2006), ^3)^Lydeard *et al*. (1996), ^4)^Simon *et al*. (1994), ^5)^Palumbi (1996) | | |

**(a)**

**(b)**

*
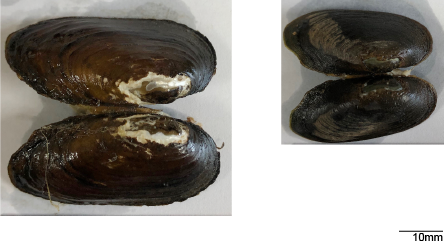
*
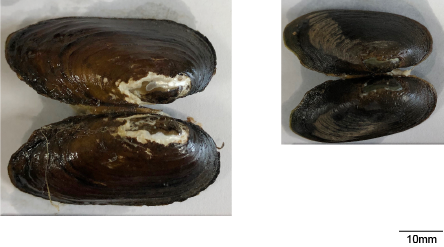
Supplementary Figure S1. The pictures showing the differences in shell shapes and sizes of (a) *Nodularia douglasiae*, and (b) *Nodularia breviconcha* collected from South Korean freshwaters. Photos by Seung Hyun Cha.

**
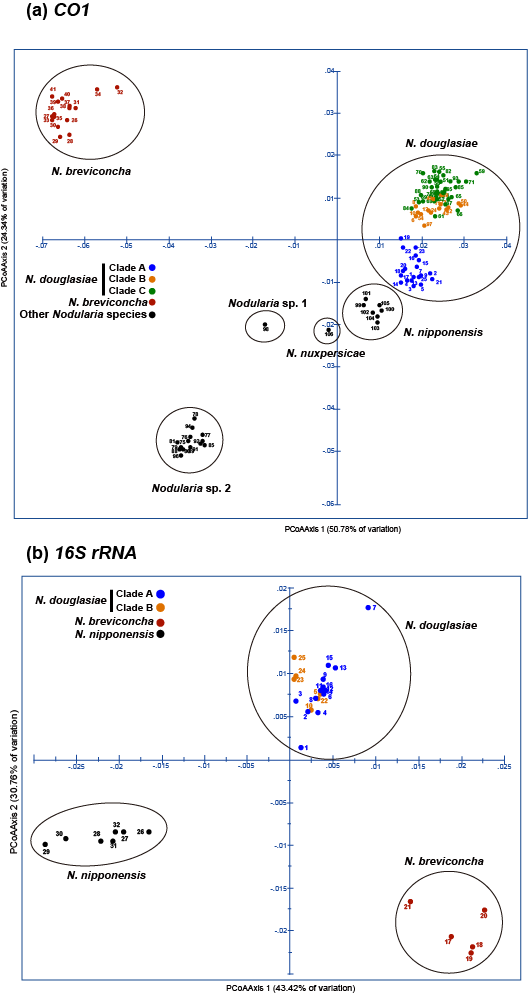
**

Supplementary Figure S2. The results of the principal coordinates analyses (PCoA) obtained with (a) 106 *CO1* haplotypes of the six *Nodularia* species and (b) 32 *16S rRNA* haplotypes of the three *Nodularia* species inhabiting East Asian countries (The haplotype information refers to Supplementary Table S5-S6).

***
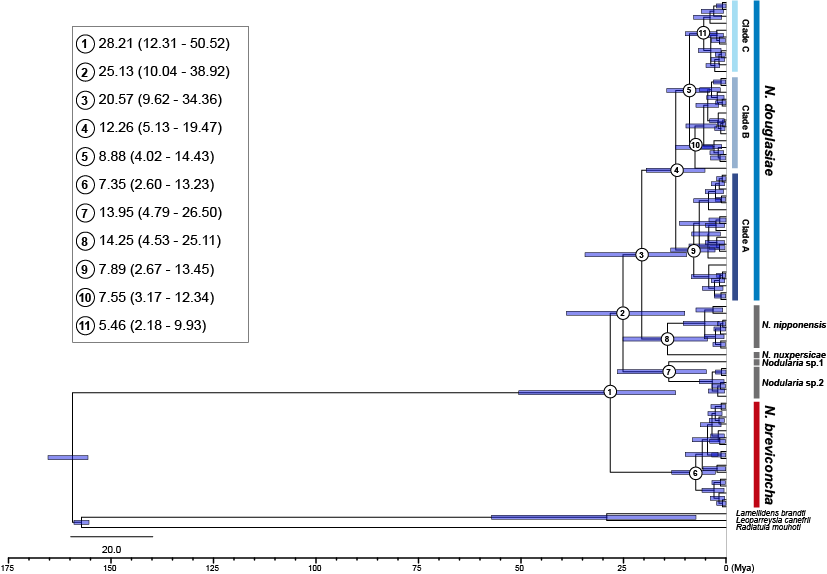
***

Supplementary Figure S3. Time-calibrated Bayesian tree reconstructed with 106 *CO1* haplotypes of six *Nodularia* species using the BEAST 2.6.0. program (The haplotype information used here is listed in Supplementary Table S5).

***
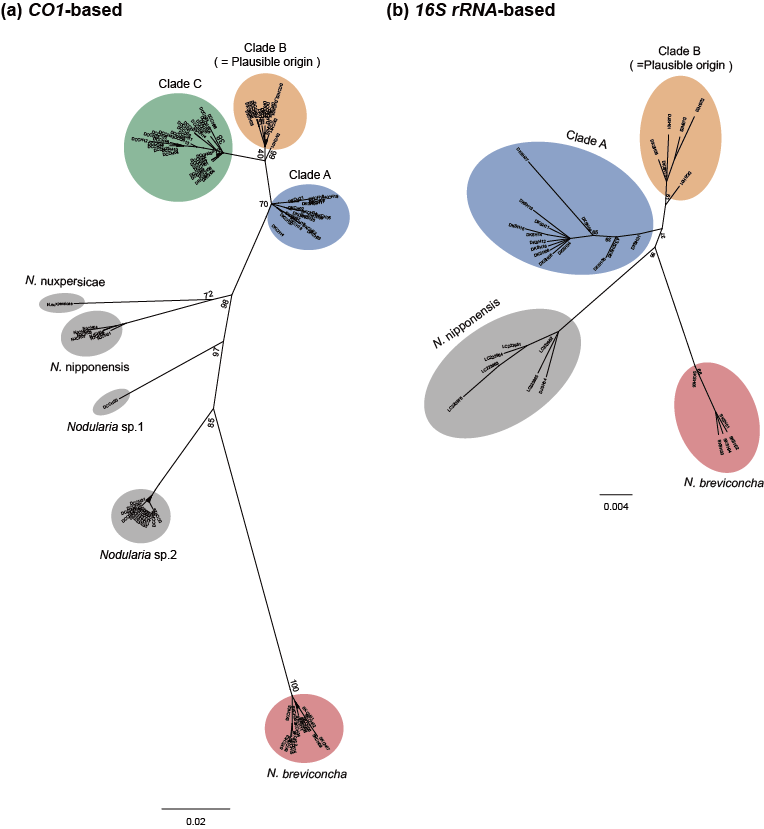
***

Supplementary Figure S4. Unrooted maximum likelihood trees showing phylogenetic relationships which was reconstructed with (a) 106 *CO1* haplotypes among the six *Nodularia* species, and (b) 32 *16S rRNA* haplotypes among the three *Nodularia* species. The Clade B might be a plausible origin of the six *Nodularia* species.
